# Design Strategies for Luminescent Titanocenes: Improving the Photoluminescence and Photostability of Arylethynyltitanocenes

**DOI:** 10.1021/acs.inorgchem.3c02712

**Published:** 2023-10-13

**Authors:** Matilda Barker, Thomas J. Whittemore, Henry C. London, Jack M. Sledesky, Elizabeth A. Harris, Tiffany M. Smith Pellizzeri, Colin D. McMillen, Paul S. Wagenknecht

**Affiliations:** † Department of Chemistry, Furman University, Greenville, South Carolina 29609, United States; ‡ Department of Chemistry and Biochemistry, Eastern Illinois University, Charleston, Illinois 61920, United States; § Department of Chemistry, Clemson University, Clemson, South Carolina 29634, United States

## Abstract

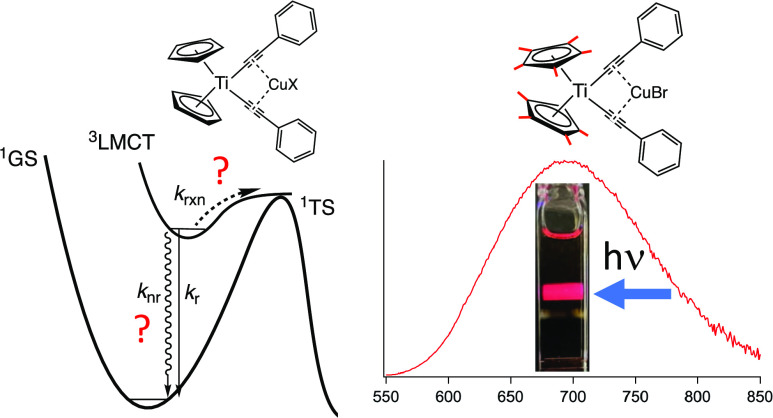

Complexes that undergo ligand-to-metal charge transfer (LMCT) to d^0^ metals are of interest as possible photocatalysts. Cp_2_Ti(C_2_Ph)_2_ (where C_2_Ph = phenylethynyl) was reported to be weakly emissive in room-temperature (RT) fluid solution from its phenylethynyl-to-Ti ^3^LMCT state but readily photodecomposes. Coordination of CuX between the alkyne ligands to give Cp_2_Ti(C_2_Ph)_2_CuX (X = Cl, Br) has been shown to significantly increase the photostability, but such complexes are not emissive in RT solution. Herein, we investigate whether inhibition of alkyne-Ti-alkyne bond compression might be responsible for the increased photostability of the CuX complexes by investigating the decomposition of a structurally constrained analogue, Cp_2_Ti(OBET) (OBET = *o*-bis(ethynyl)tolane). To investigate the mechanism of nonradiative decay from the ^3^LMCT states in Cp_2_Ti(C_2_Ph)_2_CuX, the photophysical properties were investigated both upon deuteration and upon rigidifying in a poly(methyl methacrylate) film. These investigations suggested that inhibition of structural rearrangement may play a dominant role in increasing emission lifetimes and quantum yields. The bulkier Cp*_2_Ti(C_2_Ph)_2_CuBr was prepared and is emissive at 693 nm in RT THF solution with a photoluminescent quantum yield of 1.3 × 10^–3^ (τ = 0.18 μs). Time-dependent density functional theory (TDDFT) calculations suggest that emission occurs from a ^3^LMCT state dominated by Cp*-to-Ti charge transfer.

## Abstract 2

Coordination of CuX (X = Cl, Br) into the alkyne cleft of Cp_2_Ti(C_2_Ph)_2_ drastically increases the photostability but shuts down emission in RT fluid solution. Herein, we investigate the mechanisms by which CuX results in this behavior. These investigations suggest that steric restriction may improve the photophysical properties. A sterically bulkier complex, Cp*_2_Ti(C_2_Ph)_2_CuBr, was synthesized and is visibly phosphorescent in RT THF solution (λ_max_ = 693 nm, Φ_em_ = 1.3 × 10^−3^, τ = 0.18 μs).

## Introduction

Photocatalysis driven by charge-transfer (CT) excited states in transition-metal complexes is a central area of investigation in organic synthesis^1−8^ and in the conversion of solar energy into electricity^9−11^ (dye-sensitized solar cells—DSSCs) or fuels.^12−14^ These catalysts are dominated by rare and expensive second- and third-row transition metals such as Ru and Ir.^15^ Thus, there has been significant effort over the past decade to replace these metals with earth-abundant metals.^16−21^ Significant progress has been made using complexes of Cr,^22−25^ Mo,^26,27^ W,^28,29^ Mn,^25^ Fe,^30−33^ Co,^34,35^ Ni,^36^ Cu,^37−41^ and Zn.^42,43^ One feature that complicates the use of many metals with d^1^ through d^9^ configurations is the presence of low-lying metal-centered (MC) or d–d excited states.^44,45^ Such states are typically highly distorted and have very short lifetimes. Thus, thermal access of such states provides a rapid, nonradiative decay pathway, rendering the overall excited-state lifetime too short to undergo the type of collisional energy or electron transfer necessary for photocatalysis. This is particularly a problem for first-row transition metals where the energy of the MC states is significantly lower than those of the corresponding second- and third-row transition metals.^44,45^ One particularly intriguing strategy for overcoming the problem of the MC states is to use d^0^ complexes with ligand-to-metal charge-transfer (LMCT) excited states. A handful of such d^0^ complexes that are emissive in room-temperature (RT) fluid solution from their LMCT state have been reported over the past three decades,^46−61^ with Group 3, 4, and 5 metallocenes playing a prominent role.^46−56^ More recently, such behavior has been exploited in a series of Zr^IV^ pyridine dipyrrolide complexes which are not only emissive in RT fluid solution but have also been shown to be photocatalysts.^59−61^ The corresponding Ti^IV^ complexes are not emissive.^59^ Lastly, titanocene dichloride, Cp_2_TiCl_2_, has been shown to be a photocatalyst in, for example, the reductive ring opening of epoxides,^62,63^ yet no emission is observed from Cp_2_TiCl_2_ in RT fluid solution.

**Figure 1:**
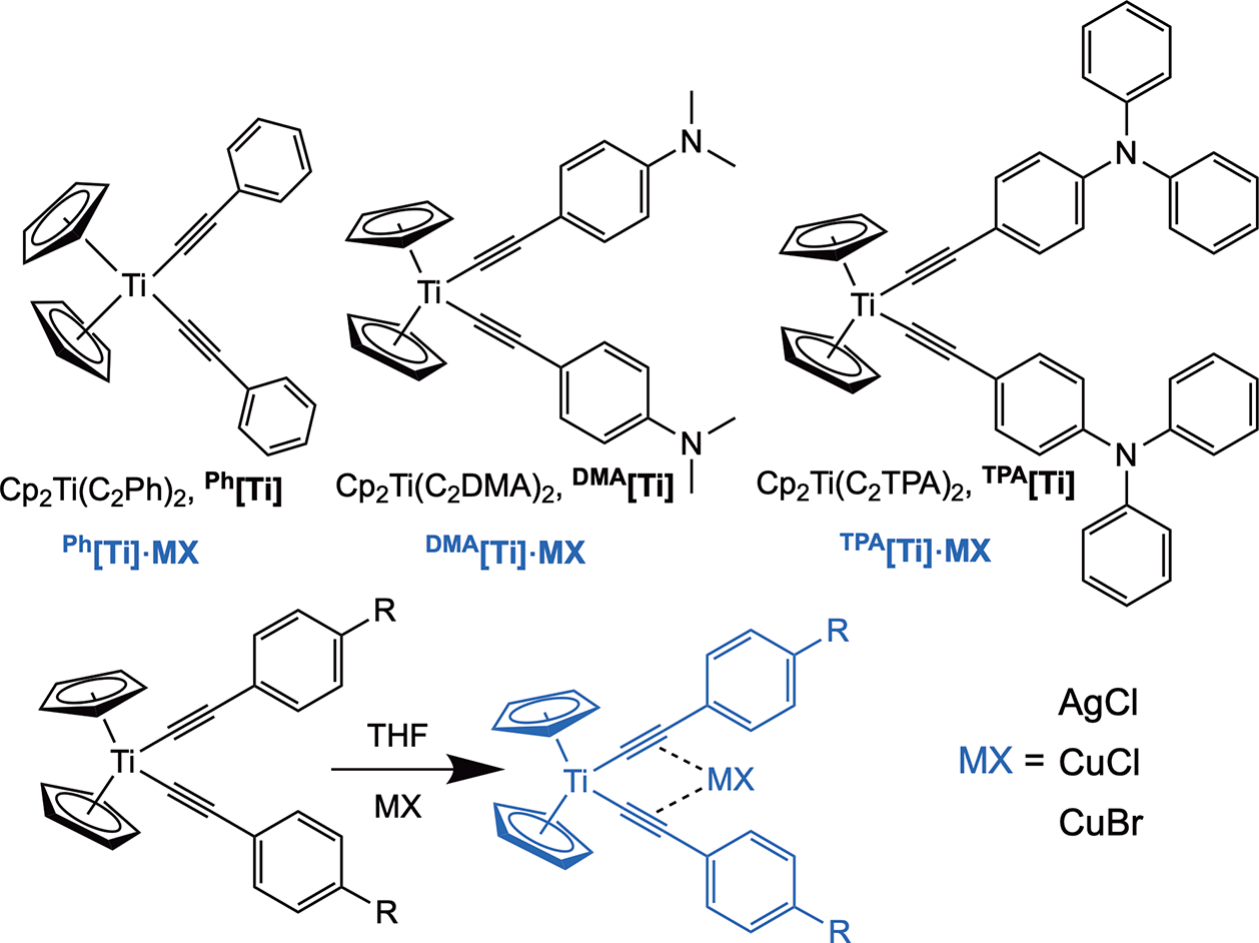
Cp_2_Ti(C_2_R)_2_ (abbreviated ^**R**^**[Ti]**) and Cp_2_Ti(C_2_R)_2_MX complexes (abbreviated ^**R**^**[Ti]MX**) discussed herein. **[Ti]** represents the Cp_2_Ti(C_2_−)_2_ core, and the superscripted R represents the aryl substituent on the alkyne.

Recently, we have been interested in the further development of Ti^IV^ complexes as possible photocatalysts (e.g., the titanocenes shown in Figure 1, where ^**R**^**[Ti]** represents Cp_2_Ti(C_2_R)_2_ and Ph, DMA, and TPA indicate the identity of R as phenyl, dimethylaniline, and triphenylamine, respectively).^64−66^ Many titanocene complexes, including the ^**R**^**[Ti]** complexes in Figure 1, have been shown to be emissive from a ^3^LMCT excited state at 77 K,^65−68^ but as alluded to above, emission in RT fluid solution from Ti^IV^ complexes has been elusive. However, we recently demonstrated that ^**Ph**^**[Ti]** is weakly emissive in RT fluid solution (λ_max_ = 574 nm), whereas the corresponding arylalkynyl titanocenes ^**DMA**^**[Ti]** and ^**TPA**^**[Ti]** are not.^66^ Furthermore, all of the ^**R**^**[Ti]** complexes investigated are photoreactive in RT solution with decomposition quantum yields (Φ_rxn_) ranging from 0.25 to 0.99. The chief organic photoproduct is an enyne, apparently formed by the coupling of two alkynyl ligands accompanied by hydrogenation of the resulting diyne.^65,66,69^

**Figure 2:**
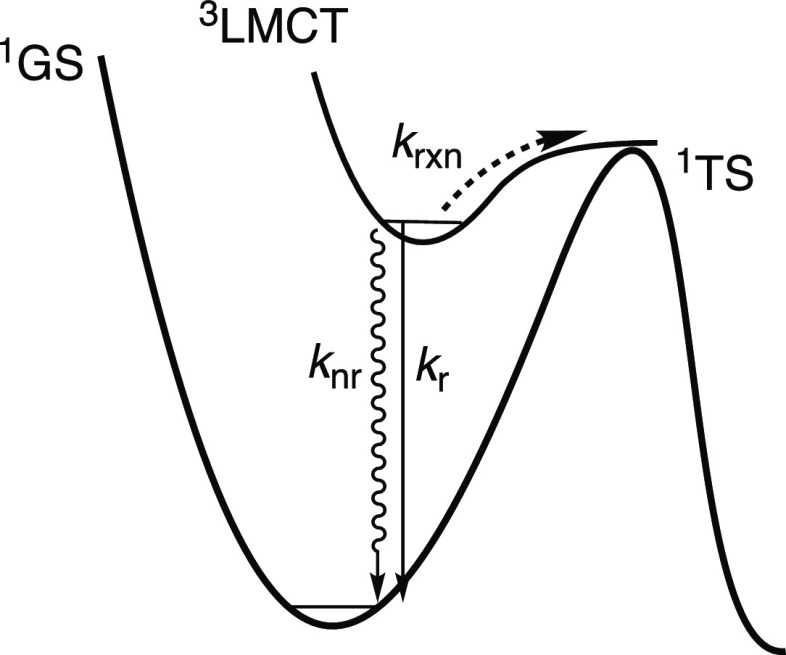
Potential well diagram showing proposed reactive and nonreactive pathways for relaxation from the ^3^LMCT state of the ^**R**^**[Ti]** and ^**R**^**[Ti]MX** complexes. The vertical axis is energy, and the horizontal axis is the nuclear coordinate.

The coordination of MX (where MX = CuBr, CuCl) into the alkyne cleft of the ^**R**^**[Ti]** complexes (Figure 1) decreased the quantum yield for photodecomposition by 2 to 3 orders of magnitude.^66^ For example, the photodecomposition quantum yield of ^**Ph**^**[Ti]** in air-saturated, room-temperature THF is 0.99, whereas the corresponding quantum yield for the CuBr complex, ^**Ph**^**[Ti]CuBr**, is 1.5 × 10^–2^. Coordination of MX into the alkyne cleft also red-shifted the ^3^LMCT emission compared to the MX-free parent complexes (measured in 77 K solvent glass). For all complexes, there was a clear trend that the photodecomposition quantum yield decreased as the emission energy decreased. A model where both emission and photodecomposition occur out of the lowest-energy ^3^LMCT state was proposed (Figure 2).^66^

Because phosphorescence in RT solution is negligible in all of these titanocenes, the quantum yield for photodecomposition is controlled by the competition between the rate constant for photodecomposition, *k*_rxn_, and the rate constant for nonradiative decay, *k*_nr_. According to this model, a lower-energy ^3^LMCT state would both (1) decrease thermal access to the transition state for photodecomposition, thus lowering *k*_rxn_, and (2) increase *k*_nr_, possibly through energy-gap law behavior. Such a model is in excellent agreement with the photodecomposition quantum yield data.^66^ Another hypothesis involved MX physically stabilizing the alkynyltitanocene through a chelate effect, a mechanism that was not explicitly tested previously. Lastly, energy-gap-law behavior was also suggested as a possible reason for the lack of room-temperature emission for all complexes except ^**Ph**^**[Ti]** and ^**Ph**^**[Ti]AgCl**.^66^ Given that ^**Ph**^**[Ti]** is the first reported Ti complex that is emissive in RT solution, it is worthwhile to further investigate both the mechanism of photodecomposition and nonradiative relaxation in order to develop design principles for titanocenes for possible use as photocatalysts and emissive complexes.

**Figure 3:**
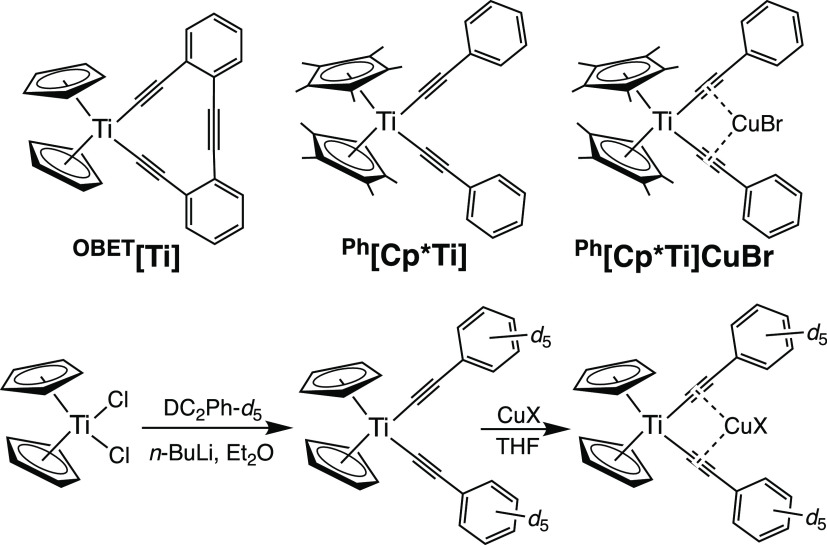
Top: Structure of the complexes investigated herein. Bottom: Synthesis of deuterated complexes, ^**Ph**^**[Ti]-*****d***_**10**_ and ^**Ph**^**[Ti]CuX-*****d***_**10**_.

Herein, we report an additional investigation of the photodecomposition mechanism by measuring the decomposition quantum yield of Cp_2_Ti(OBET), ^**OBET**^**[Ti]** (OBET = *o*-bis(ethynyl)tolane), where physical constraint is imposed by an additional alkynyl linkage between the two phenylethynyl ligands (Figure 3, top). We demonstrate that this rigidification has little impact on the photodecomposition quantum yield (compared with ^**Ph**^**[Ti]**), an observation that is consistent with the hypothesis that MX binding stabilizes the excited state toward decomposition through lowering the energy of the ^3^LMCT excited state rather than through rigidification.

Herein, we also report an investigation of the mechanism for nonradiative decay using a classic method to investigate energy-gap law behavior, i.e., investigation of the impact of deuteration on the photophysical properties.^70,71^ The synthesis and photophysical characterization of ^**Ph**^**[Ti]-*****d***_**10**_, as well as the corresponding CuX complexes (X = Cl or Br), ^**Ph**^**[Ti]CuX-*****d***_**10**_ (Figure 3, bottom) that have been perdeuterated at the Ph rings are presented. Little-to-no impact of deuteration on the excited-state lifetime was observed, suggesting the possibility of an alternate mechanism for nonradiative deactivation such as crossing between potential-energy surfaces. The activation barrier for such crossing can be impacted by rigidification to the extent that rigidification affects both the excited-state energy and the displacement of that potential-energy surface along the nuclear coordinate. Thus, the impact of rigidification of ^**Ph**^**[Ti]CuBr** in poly(methyl methacrylate) (PMMA) film was also investigated. Although ^**Ph**^**[Ti]CuBr** is not emissive in solution, it is emissive in PMMA films, suggesting that the crossing between potential-energy surfaces dominates nonradiative decay and is facilitated by the structural reorganization in the excited state. This further suggests that steric restriction may increase the luminescent quantum yield. In order to test this, an analogue of ^**Ph**^**[Ti]CuBr**, where the Cp rings were replaced with pentamethylcyclopentadienyl, ^**Ph**^[Cp*Ti]CuBr (Figure 3, top), was prepared. This complex is luminescent in RT fluid solution (Φ_P_ = 1.3 × 10^–3^), in contrast to ^**Ph**^**[Ti]CuBr** which is not. Furthermore, ^**Ph**^[Cp*Ti]CuBr has a relatively small photodecomposition quantum yield (1.5 × 10^–2^), similar to that of ^**Ph**^**[Ti]CuBr**.

## Experimental Section

### Materials and Methods

The complexes ^**Ph**^**[Ti]-*****d***_**10**_, and ^**Ph**^**[Ti]CuX-*****d***_**10**_, were prepared according to literature procedures,^64−66,72^ modified by using *d*_6_-phenylacetylene (Figure 3). ^**OBET**^**[Ti]** was prepared according to the literature procedure.^73^ Phenylacetylene-*d*_6_ was obtained from CDN Isotopes; 2,2′-dibromodiphenylbenzene (precursor to the OBET ligand) was obtained from Biosynth; bis(pentamethylcyclopentadienyl)titanium dichloride was obtained from Acros Organics. ^1^H and ^13^C NMR spectra were obtained using a JEOL-500 spectrometer. UV–vis absorption spectra were recorded using a Cary-50 spectrophotometer. Infrared spectra were obtained using a PerkinElmer Spectrum Two FT-IR spectrometer with a UATR attachment. Emission spectra were recorded using a Horiba Fluorolog-3 Spectrofluorometer equipped with either an FL-1013 liquid nitrogen dewar assembly or J-1933 solid-sample holder. All emission spectra were corrected for the response factor of the R928 photomultiplier tube. Relative solution-state photoluminescence quantum yields for ^**Ph**^[Cp*Ti]CuBr in tetrahydrofuran (THF) were determined using a Ru(bpy)_3_^2+^ standard in air-saturated CH_3_CN (Φ_PL_ = 0.018),^74^ with solutions that were absorbance-matched at the excitation wavelength across several absorption values and two different excitation wavelengths (430 and 450 nm). Emission spectra in RT solution were additionally corrected with blank subtraction. Emission lifetimes were measured by using a Photon Technology International (PTI) GL-3300 pulsed nitrogen laser fed into a PTI GL-302 dye laser as the excitation source. The resulting data set was collected on an OLIS SM-45 EM fluorescence lifetime measurement system using a Hamamatsu R928 photomultiplier tube fed through a variable feedthrough terminator into a LeCroy Wavejet 352A oscilloscope and analyzed using OLIS Spectral Works. PMMA films were prepared either by doctor blading or drop-casting a solution of PMMA (*M*_W_ ∼ 120,000) and the analyte in CH_2_Cl_2_ solution. Elemental analyses were performed by Midwest Microlabs.

### Syntheses

#### Cp*_2_Ti(C_2_Ph)_2_, ^**Ph**^[Cp*Ti]

To an oven-dried 50 mL two-neck round-bottom flask under a positive pressure of argon were added THF (20 mL) and phenylacetylene (0.56 mL, 5.1 mmol, 4.0 equiv). After the pale-yellow solution was cooled in a dry ice/acetone bath for 10 min, *n*-butyllithium (2.5M, 2.2 mL, 5.5 mmol, 4.3 equiv) was added. After the mixture was stirred for 10 min, the flask was removed from the bath and stirred for an additional 10 min. Cp*_2_TiCl_2_ (500 mg, 1.28 mmol, 1.0 equiv) was added, and the mixture was stirred at room temperature in the absence of light for 3 h. The solvent was removed using rotary evaporation, and the resulting solid was loaded onto a silica gel column (2 × 15 cm) and eluted using a 5% mixture of triethylamine in CH_2_Cl_2_. The red band was collected and the solvent was removed using rotary evaporation. Hexanes (30 mL) was added and the mixture was sonicated and then chilled to −20 °C for 40 min. The solid was collected using vacuum filtration and dried under vacuum (502 mg, 75.1%). UV–Vis (THF) λ_max_ (ε); 528 sh (1930), 467 (3590), 384 (8660), 349 (10300), 261 (47300). ^1^H NMR (500 MHz, CDCl_3_) δ 7.32 (m, 4H, *ortho*-CH), 7.23 (m, 4H, *meta*-CH), 7.14 (m, 2H, *para*-CH), 2.07 (s, 30H, CH_3_); ^13^C {^1^H} NMR (125 MHz, CDCl_3_) δ 161.7, 130.9, 128.1, 127.1, 126.1, 123.5, 122.0, 13.3. Anal. Calcd (found) for C_36_H_40_Ti: C, 83.06 (83.37); H, 7.74 (7.97). IR (neat, ATR) ν_C≡C_ = 2069 cm^–1^.

#### Cp*_2_Ti(C_2_Ph)_2_CuBr, ^**Ph**^[Cp*Ti]CuBr

To an oven-dried 50 mL two-neck round-bottom flask under a positive pressure of argon were added Cp*_2_Ti(C_2_Ph)_2_ (100 mg, 0.192 mmol) and CuBr (55 mg, 0.384 mmol, 2.0 equiv). THF (16 mL) was then added and the mixture was allowed to stir at room temperature in the absence of light for 2 h. The reaction mixture was then vacuum-filtered to remove unreacted CuBr, and the solvent was removed using rotary evaporation. THF (3 mL) was added and the mixture was sonicated and then chilled to −20 °C for 1 h. The solid was collected using vacuum filtration and was then washed with hexanes (10 mL), and the solid was dried under vacuum yielding a red solid (70 mg, 55%). UV–Vis (THF) λ_max_ (ε); 465 sh (4410), 381 (8840), 264 (46700). ^1^H NMR (500 MHz, CDCl_3_) δ 7.69 (m, 4H, *ortho*-CH), 7.35 (m, 4H, *meta*-CH), 7.29 (m, 2H, *para*-CH), 1.98 (s, 30H, CH_3_); ^13^C {^1^H} NMR (125 MHz, CDCl_3_) δ 149.4, 134.2, 132.3, 128.4, 128.2, 124.8, 121.7, 13.1. Anal. Calcd (found) for C_36_H_40_TiCuBr•H_2_O: C, 63.40 (63.51); H, 6.21 (6.09). IR (neat, ATR) ν_C≡C_ was not observed.

### Determination of Photodecomposition Quantum Yields

For ^**OBET**^**[Ti]**, photolyses were performed using a Rayonet RPR-100 Photochemical Reactor with four 419 nm bulbs (RPR-4190). Photon flux (1.1 × 10^–7^ mol/s) was determined using ferrioxalate actinometry,^75^ using the same sample volume (2.25 mL) and cell geometry used for the photolyses. The contents of the cuvette were stirred continuously during the period of the photolysis. The desired analyte (8–10 mg) and a phenanthroline internal standard were dissolved in 3.0 mL of C_6_D_6_, and a 0.75 mL aliquot was transferred to an NMR tube and protected from light. The concentration used ensured complete absorption of the incident radiation. The remaining 2.25 mL sample in a quartz cuvette was photolyzed for 100 s, after which another 0.75 mL aliquot was transferred to an NMR tube. The two samples were then analyzed by ^1^H NMR using 64 scans and a 6 s relaxation delay to ensure quantitative integrals, and the internal standard was used to determine the amount of analyte that decomposed. For ^**Ph**^[Cp*Ti]CuBr, photodecomposition quantum yields (Φ_rxn_) were performed using the method previously published for the ^**R**^**[Ti]MX** complexes,^66^ but using a 428 nm diode laser (RMPC Laser) as the excitation source. All reported Φ_rxn_ values are averages of at least three replicates.

### Computational Methods

Gaussian 16^76^ was used for all density functional theory (DFT) and time-dependent DFT (TDDFT) calculations. For each computational model, the geometry was optimized and the structure was checked to be a minimum based on the frequency calculation. GaussView, version 6.32^77^ was used for all orbital imaging. Mulliken population analysis was performed using GaussSum3.^78^ For ^**OBET**^**[Ti]**, the computational model used the functional MN15,^79^ and the basis set LANL2DZ^80^ for both optimization and TDDFT, which was demonstrated to accurately model geometry and optical spectra for the ^**R**^**[Ti]** complexes.^65^ For ^**Ph**^[Cp*Ti]CuBr, TDDFT used the same MN15/LANL2DZ model on a geometry that was optimized using the functional B3LYP^81^ and the basis set 6-311+G(d).^82^ This model was demonstrated to accurately model the ^**R**^**[Ti]CuX** complexes.^66^ Because all spectroscopic data reported herein are recorded in THF or 2-methyltetrahydrofuran, all calculations employed a Tomasi polarizable continuum model assigned the dielectric constant for THF.^83^

### X-ray Crystallography

Single crystals of ^**Ph**^[Cp*Ti]CuBr were grown by vapor diffusion of Et_2_O into a solution of the complex in THF with 5% triethylamine. Single-crystal X-ray diffraction data were collected at 100 K by using a Bruker D8 Smart Apex 2 diffractometer with Cu Kα radiation (λ = 1.5406 Å). Data collection, data processing (SAINT), scaling, and absorption correction (SADABS, multiscan) were performed using the Apex 3 software suite.^84^ Space group determination (XPREP), structure solution by intrinsic phasing (SHELXT), and structure refinement by full-matrix least-squares techniques on *F*^2^ (SHELXL) were performed using the SHELXTL software package.^85^ All nonhydrogen atoms were refined anisotropically. Hydrogen atoms attached to carbon atoms were placed in calculated positions by using appropriate riding models. Disorder of the Cp* ligands and phenyl rings on the phenylethynyl ligands was modeled in separate parts, and the occupancies of the major and minor contributing components were freely refined (52:48 for Cp* and 53:47 for the phenyl rings). Appropriate similarity restraints on the bond lengths and anisotropic displacement parameters were used to maintain chemically reasonable similarities between the disordered parts. The Flack parameter of 0.000(18) supports refinement in the correct absolute structure. Crystallographic data are provided in the Supporting Information, Table S1. Crystallographic data are available in CIF form through the Cambridge Crystallographic Data Centre, CCDC deposition number 2286856.

## Results and Discussion

### Synthesis and Characterization

All syntheses were performed under an inert atmosphere, but the titanocene products are air-stable and can be handled under ambient conditions. The syntheses of ^**Ph**^**[Ti]** and ^**Ph**^**[Ti]CuX** have been reported previously,^64−66,72^ and herein a similar procedure was followed using *d*_6_-phenylacetylene to yield ^**Ph**^**[Ti]-*****d***_**10**_ and ^**Ph**^**[Ti]CuX-*****d***_**10**_ (Figure 3, bottom). The ^1^H NMR spectra are identical to those of the corresponding protio complexes but with the absence of the phenyl protons (Figures S1–S3). ^**OBET**^**[Ti]** was prepared according to the literature procedure.^73^ UV–vis spectra with molar absorptivity, emission spectra, and quantum yields for the photodecomposition of ^**OBET**^**[Ti]** were not previously reported and are presented herein. ^**Ph**^[Cp*Ti] was prepared according to the procedures previously published for the ^**R**^**[Ti]** complexes,^64^ where the corresponding titanocene dichloride is treated with the appropriate lithiated alkyne. For ^**Ph**^[Cp*Ti], however, yields using diethyl ether as solvent were very low. Yields reported herein are in a THF solvent. The NMR data support the structure (Figure S4). The synthesis of ^**Ph**^[Cp*Ti]CuBr utilized the same procedure previously reported for ^**Ph**^**[Ti]CuBr**, i.e., ^**Ph**^[Cp*Ti] was treated with CuBr in THF solvent.^66^ However, the purification method used for all previous ^**R**^**[Ti]MX** complexes, i.e., elution down a silica gel column using 5% triethylamine in CH_2_Cl_2_ as the eluent, appeared to introduce a minor impurity (∼10% by integration) that appeared as an additional doublet at 7.72 ppm in the ^1^H NMR (Figure S5) that was not present in the crude material. Changing the eluent to THF did not improve the purity. Thus, purification by column chromatography was replaced with recrystallization from THF, resulting in a pure product (Figure S5). The photobehavior was not affected by the purification method.

**Figure 4:**
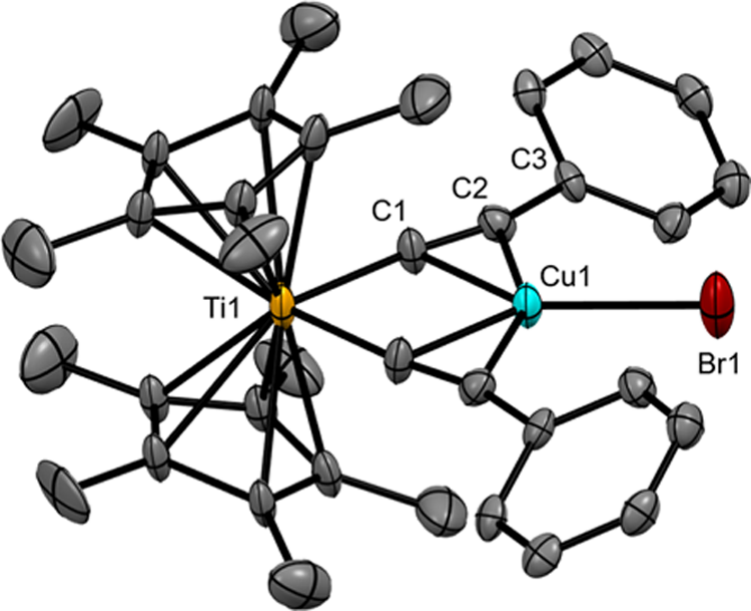
Structure of the ^**Ph**^[Cp*Ti]CuBr complex shown as 30% probability ellipsoids. Atoms pictured are the majority-occupied disordered arrangements of the phenyl and Cp* rings. Hydrogen atoms have been omitted for clarity.

The ^**Ph**^[Cp*Ti]CuBr complex crystallizes in the tetragonal space group *P*4_3_2_1_2 with Z = 4 (Figure 4, Table S1, Figures S6–S8). Half of the molecule is unique, with the Ti, Cu, and Br atoms sitting on special positions having 2-fold rotational symmetry. The phenylethynyl ligands create a binding pocket through their coordination to Ti (Ti–C = 2.080(8) Å) having a C–Ti–C bond angle of 88.0(4)°. The C≡C triple bond has a length of 1.227(11) Å, similar to those in the related ^**Ph**^**[Ti]CuBr** complex,^66^ with Ti–C≡C and C≡C–C angles of 172.0(7) and 160.1(9)°, respectively. The Cu atom sits in the binding pocket at an appropriate distance to coordinate both carbon atoms of both alkynyl ligands (2.069(8) and 2.222(8) Å) and maintains a distance of 2.976(2) Å from Ti. The Cu–Br bond length of 2.3087(17) Å is similar to that in ^**Ph**^**[Ti]CuBr**.^66^ The Cp* ligands are inclined from the central Ti-alkyne–Cu-Br plane at 18.8(11)° and create a centroid-Ti-centroid angle of 141.6(11)° with Ti-centroid distances of 2.04(2) Å. This centroid-Ti-centroid angle is significantly larger than the corresponding ^**Ph**^**[Ti]CuBr** complex (134.2°).^66^ The Cp* ligands consist of a planar Cp core with the methyl substituents slightly out of this plane, deviating from the plane by an average of 0.20(2) Å to a maximum of 0.30(2) Å. This expansion of the centroid-Ti-centroid angle (relative to the Cp analogue) and out-of-plane deviations of the methyl substituents is indicative of methyl–methyl contacts between the two rings and was first reported for titanocenes by Bercaw’s group.^86^ Chiefly, for Cp*_2_TiCl_2_, the Cp*(centroid)–Ti-Cp*(centroid) bond angle has been described to result from a balance of minimizing methyl–methyl and methyl–chlorine nonbonding interactions,^86^ an indication of the steric restriction that might result in such a system.

The Cp* ligands and phenyl rings exhibit disorder. The Cp* disorder is indicative of rotational freedom about its centroid coordination axis with Ti. The phenyl ring disorder suggests that the phenylethynyl ligands have freedom to exist as rotamers relative to the central Ti-alkyne–Cu-Br plane. The occupancy distribution of these disordered rotamers is about equal, with one orientation inclined at 31.1(7)° to the Ti-alkyne–Cu-Br plane, and the other orientation nearly coplanar with the Ti-alkyne–Cu-Br plane, inclined at 1.6(13)° (Figure S7). For the corresponding Cp complex,^66^ the phenyl rings are significantly more perpendicular to the Ti-alkyne–Cu-Br plane (Figure S8), again indicative of the structural constraint imposed by the Cp* ligands.

### ^**OBET**^**[Ti]**: Investigating the Impact of Molecular Rigidification on Photochemistry

#### Previous Research

**Figure 5:**
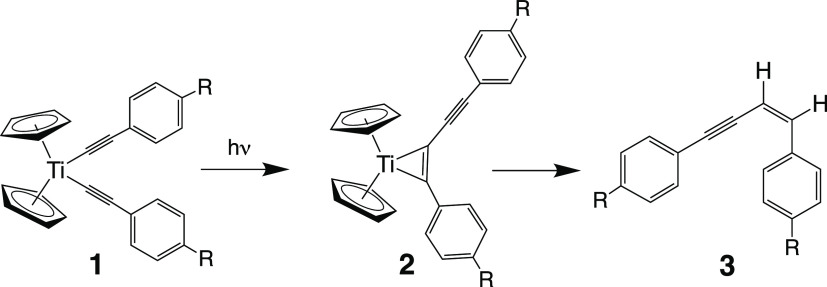
Organic decomposition product, **3**, formed from photolysis of **1**, along with the proposed intermediate, **2**.

Previous investigations demonstrated that the chief organic photoproduct from photolysis of the ^**R**^**[Ti]** complexes was an enyne (**3** in Figure 5), likely from reductive elimination of a butadiyne followed by reduction of that diyne.^65,66,69^ Butadiynes are also known to coordinate to give titanacyclopropenes, and **2** (Figure 5) as well as its titanacyclocumulene form have been suggested as likely intermediates.^87−89^ Clearly, there must be a rearrangement that allows for the formation of a new C–C bond. Previous research has also shown that the optimized ^3^LMCT states for ^**R**^**[Ti]** complexes (**1** in Figure 5) have compressed C–Ti–C bond angles and elongated Ti–C bonds, which may facilitate the formation of the C–C bond.^65^ Thus, one hypothesis of how the coordination of MX stabilizes the ^**R**^**[Ti]MX** complexes toward photodecomposition was the restriction of such excited-state rearrangement. Thus, the photophysics and photochemistry of ^**OBET**^**[Ti]** (Figure 3) were investigated to determine the impact of molecular rigidity.

#### Photophysical Characterization

**Figure 6:**
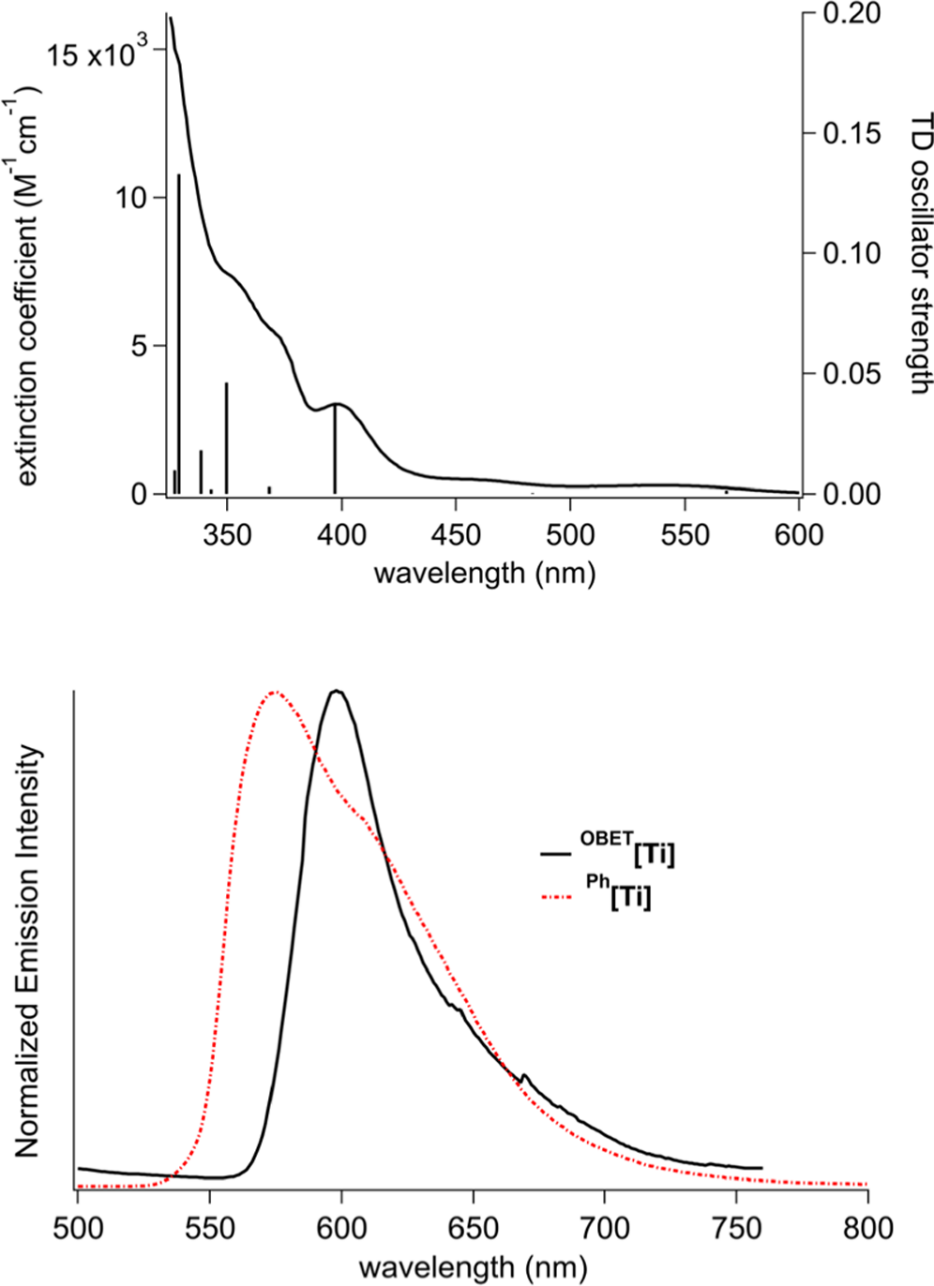
Top: Absorption spectrum of ^**OBET**^**[Ti]** in RT THF solution overlaid with TDDFT predicted vertical transitions (MN15/LANL2DZ//MN15/LANL2DZ). Bottom: Emission spectra of ^**OBET**^**[Ti]** (λ_ex_ = 399 nm) and ^**Ph**^**[Ti]** (λ_ex_ = 400 nm) in 2-methyltetrahydrofuran glass at 77 K.

**Figure 7:**
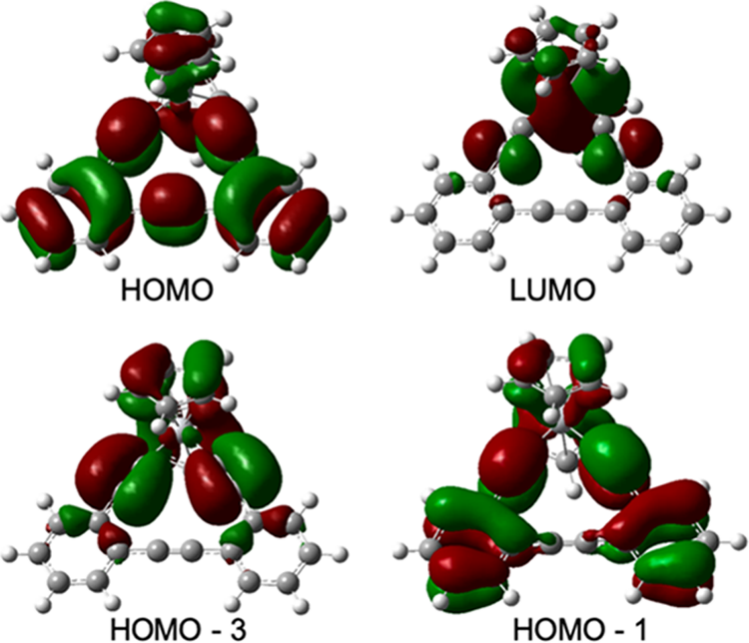
Frontier orbitals involved in lowest-energy transitions for ^**OBET**^**[Ti]** (MN15/LANL2DZ).

The absorption spectrum of ^**OBET**^**[Ti]** in THF (Figure 6) shows weak absorption bands at 541 and 460 nm. Based on TDDFT, the lowest-energy absorption is dominated by a highest occupied molecular orbital (HOMO) to lowest unoccupied molecular orbital (LUMO) transition and the next lowest-energy absorption is dominated by HOMO – 1 to LUMO (both TD and DFT using MN15/LANL2DZ, Figure 7, Charts S1 and S2). A stronger band at 400 nm (ε = 3010 M^–1^ cm^–1^) is dominated by HOMO – 3 to LUMO. All three transitions can be ascribed to OBET-to-Ti LMCT with some Cp-to-Ti LMCT character. The analogous LMCT absorption for ^**Ph**^**[Ti]** occurs at 417 nm (ε = 11,700 M^–1^ cm^–1^). One interesting feature is that the TDDFT predicted absorption spectrum for ^**OBET**^**[Ti]** in THF using the optimized geometry accurately modeled the absorption spectrum, whereas for ^**Ph**^**[Ti]**, the spectrum could only be modeled by averaging the contribution from several rotamers involving rotation of the phenyl rings.^65^ Such rotamers are not possible with the OBET ligand constraint in ^**OBET**^**[Ti]**.

The emission spectrum of ^**OBET**^**[Ti]** in 77 K solvent glass is narrower and red-shifted from that of ^**Ph**^**[Ti]** (Figure 6, Table 1). The relative breadth of the emission spectrum of ^**Ph**^**[Ti]** suggests that this spectrum is also composed of a sum of contributions from rotamers that once again is not possible for ^**OBET**^**[Ti]**. The excited state lifetime of ^**OBET**^**[Ti]** in 77 K solvent glass is 3.6 ± 0.2 ms (Figure S9, Table 1), on the same order of magnitude as that for ^**Ph**^**[Ti]**.^66^ Like ^**Ph**^**[Ti]**, ^**OBET**^**[Ti]** is also weakly emissive in RT fluid solution. The excitation spectra match the UV–vis spectra, indicating that the emission is not due to an impurity (Figure S10). The lowest-energy triplet is dominated by HOMO to LUMO, OBET-to-Ti LMCT character, indicating that the lowest-energy excited state is of ^3^LMCT origin (Figure 7).

**Table 1:** Emission Data at 77 K^a^

	λ_max_ (nm)	τ_protio_^b^	τ_deuterio_^b^
^OBET^[Ti]	598	3.6 ms	
^Ph^[Ti]	575^c^	9.17 ms	9.11 ms
^Ph^[Ti]CuBr	715	35.8 μs	37.4 μs
^Ph^[Ti]CuCl	706	21.0 μs	22.7 μs

a Emission spectra and excited-state lifetimes recorded in 2-methyltetrahydrofuran.

b Standard deviation, 4%.

c From ref (65).

#### Photodecomposition Quantum Yields

Previous investigations of Φ_rxn_ for photodecomposition of ^**Ph**^**[Ti]**, ^**DMA**^**[Ti]**, and ^**TPA**^**[Ti]** relied on quantification using UV–vis spectroscopy in THF solvent.^65^ In such cases, the absorbance for the decomposition products approached zero at the wavelength used for the determination of concentration. For the decomposition of ^**OBET**^**[Ti]**, the absorbance did not go to zero at such a wavelength, hampering the determination of concentration. Thus, ^1^H NMR spectroscopy in C_6_D_6_ using phenanthrene as an internal standard was used to monitor the disappearance of the starting material (Figure S11). Under these conditions, Φ_rxn_ for decomposition of ^**Ph**^**[Ti]** under both aerated conditions (air, Table 2) and argon purged conditions (Ar, Table 2) compare reasonably well with those previously reported in THF solvent using UV–vis to monitor decomposition. The corresponding decomposition quantum yields for ^**OBET**^**[Ti]** are approximately 60% of those for ^**Ph**^**[Ti]** under the same conditions (Table 2). Recall that photodecomposition quantum yields of ^**R**^**[Ti]** complexes decreased by 2–3 orders of magnitude upon coordination of CuX. In a relative sense, the quantum yield of photodecomposition of ^**OBET**^**[Ti]** is not significantly lowered relative to ^**Ph**^**[Ti]**. This suggests that the impact of CuX coordination is not dominated by its ability to physically restrain excited-state distortion. It is also noteworthy that the ^3^LMCT state energy is slightly lower for ^**OBET**^**[Ti]** than for ^**Ph**^**[Ti]** and thus the slightly lower Φ_rxn_ for ^**OBET**^**[Ti]** is also consistent with the model discussed in Figure 2, wherein a lower excited-state energy renders the transition state for photodecomposition less accessible.

**Table 2:** Quantum Yields^a^ for Photodecomposition of ^**Ph**^**[Ti]** and ^**OBET**^**[Ti]**

	^**Ph**^**[Ti]**, THF^b^	^**Ph**^**[Ti]**, C_6_D_6_	^**OBET**^**[Ti]**, C_6_D_6_
Ar	0.65	0.53	0.30
air	0.99	0.69	0.44

a Estimated error, ±20%

b From ref (65).

Lastly, the ^1^H NMR spectrum of a solution of ^**OBET**^**[Ti]** in C_6_D_6_ that was photolyzed to complete decomposition showed significant activity between 5 and 9 ppm, suggesting numerous aryl and alkene protons (Figure S12). Based on the steric constraint within the OBET ligand, an intramolecular C–C bond-forming reaction is unlikely, suggesting possible oligomer formation, consistent with the ^1^H NMR spectrum. However, we were unable to identify simple dimers and trimers using mass spectrometry, perhaps due to the lack of oligomer ionization or due to more complex decomposition products.

### Investigating the Mechanism of Nonradiative Decay

#### Impact of Deuteration on Photophysics

Though coordination of CuX improves the photostability, likely by lowering the energy of the excited state, none of the ^**R**^**[Ti]CuX** complexes are emissive in RT fluid solution. The hypothesis that the energy-gap law is partly responsible for the photophysical behavior of the ^**R**^**[Ti]** and ^**R**^**[Ti]MX** complexes implies a weak coupling limit where the excited-state potential well is not significantly displaced along the nuclear coordinate relative to the ground-state potential well. This results in nested potential wells where radiationless deactivation is facilitated through high-energy X-H vibrations acting as the acceptor modes.^71^ For example, in a series of Cp*Ta(OAr)_4_ (where OAr = OPh, *p*-OC_6_H_4_OMe, *p*-OC_6_H_4_-*i*-Pr, OC_6_F_5_, and OC_6_Cl_5_), none of the complexes with C–H bonds in the aryl substituent were emissive at 77 K, whereas those with fully substituted C–H bonds were emissive. It was suggested that these complexes are emissive from their OAr-to-Ta ^3^LMCT state and that the C–H bonds may be involved in facile nonradiative processes.^48^ When such C–H vibrations serve as acceptor modes, deuteration decreases the vibrational overlap and decreases the rate constant for nonradiative relaxation.^71^ For ^**Ph**^**[Ti]**, the emissive excited state has been characterized as phenylethynyl-to-titanium LMCT in character; thus, the impact of deuteration of the phenylethynyl ligand on the emission lifetime was investigated. Effects of deuteration on lifetime will be most pronounced at 77 K, where competing mechanisms (i.e., photodecomposition or activated crossing) for nonradiative relaxation are minimized. The lifetimes of absorbance-matched samples of ^**Ph**^**[Ti]** (9.17 ms) and ^**Ph**^**[Ti]-*****d***_**10**_ (9.11 ms, Figure S13, Table 1) in 2-methyltetrahydrofuran glass at 77 K are identical within experimental error, indicating that deuteration of the phenyl ring does not impact the rate constant for nonradiative decay.

According to the energy-gap law, ^**Ph**^**[Ti]** would be expected to have the smallest value for *k*_nr_ among the arylalkynyl titanocenes previously investigated (Figure 1), because it has the highest-energy ^3^LMCT state (λ_em_ = 575 nm, 77 K glass).^66^ Thus, the deuterated versions of the corresponding ^**Ph**^**[Ti]CuX** complexes (X = Br, λ_em_ = 715 nm; X = Cl, λ_em_ = 706 nm, 77 K glass)^66^ were also investigated. As mentioned above, these complexes are not emissive in RT fluid solution. The isotopically labeled versions, ^**Ph**^**[Ti]CuX-*****d***_**10**_, also showed no emission in RT fluid solution. Thus, the complexes were investigated at 77 K where both the deuterated and undeuterated analogues are brightly emissive. For each complex, there was a very modest increase in lifetime upon deuteration that is not significantly larger than the standard error (Table 1). Chiefly, for ^**Ph**^**[Ti]CuBr** the lifetime increased from 35.8 to 37.4 μs upon deuteration (Figure S14). For ^**Ph**^**[Ti]CuCl** the lifetime increased from 21.0 to 22.7 μs upon deuteration (Figure S15). Lifetime measurements were made on absorbance-matched samples and identical instrument settings to minimize error, but multiple measurements on several samples suggest up to a 4% standard deviation, suggesting at most a negligible increase of excited-state lifetime upon deuteration.

**Figure 8:**
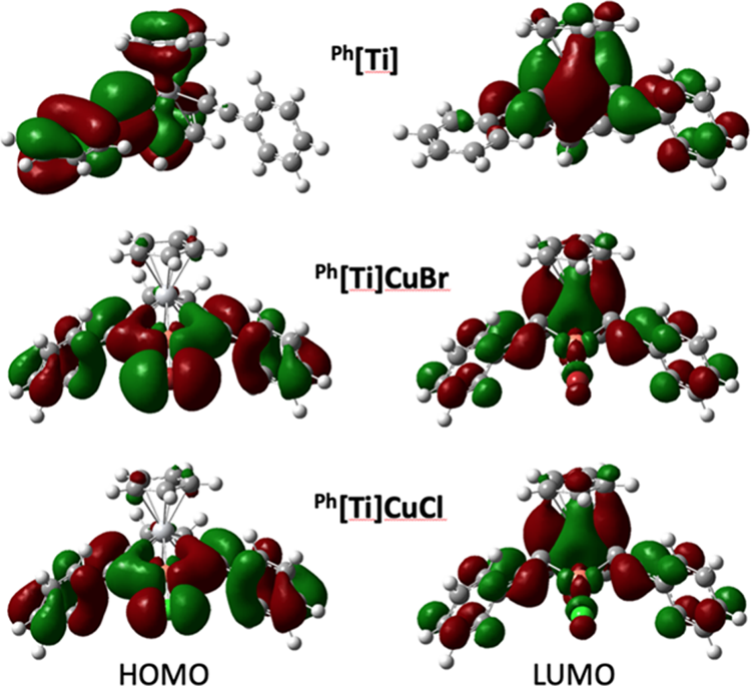
HOMO (left) and LUMO (right) images (isovalue = 0.02) for ^**Ph**^**[Ti]**, ^**Ph**^**[Ti]CuBr**, and ^**Ph**^**[Ti]CuCl** at the B3LYP/6-311+G(d) level of theory.^65,66^

One possible explanation for a lack of sensitivity to deuteration is that the excited-state transition does not significantly involve orbitals on the phenyl rings. However, previous computational research has demonstrated that emission in ^**Ph**^**[Ti]** occurs from a ^3^LMCT state where electron density has been promoted from the HOMO (dominated by orbitals on the C_2_Ph ligand) to LUMO (dominate by Ti d-orbital character). Coordination of CuBr to give ^**Ph**^**[Ti]CuBr** results in a triplet emissive state that still retains some such LMCT character but is dominated by CuBr to Ti charge-transfer character. The degree of CuX to Ti charge-transfer character for ^**Ph**^**[Ti]CuCl** is diminished so that the emissive state is again dominated by ^3^LMCT character (Figure 8).^66^ Thus, computational evidence suggests that there is a significant involvement of orbitals on the phenyl rings in the emissive excited state for each complex. Regardless, the lack of sensitivity to deuteration is a negative result and thus does not preclude nonradiative decay from being dominated by energy-gap law behavior. However, the implication still led us to investigate other mechanisms for nonradiative relaxation.

#### Impact of a Rigid Matrix on Photophysics

**Figure 9:**
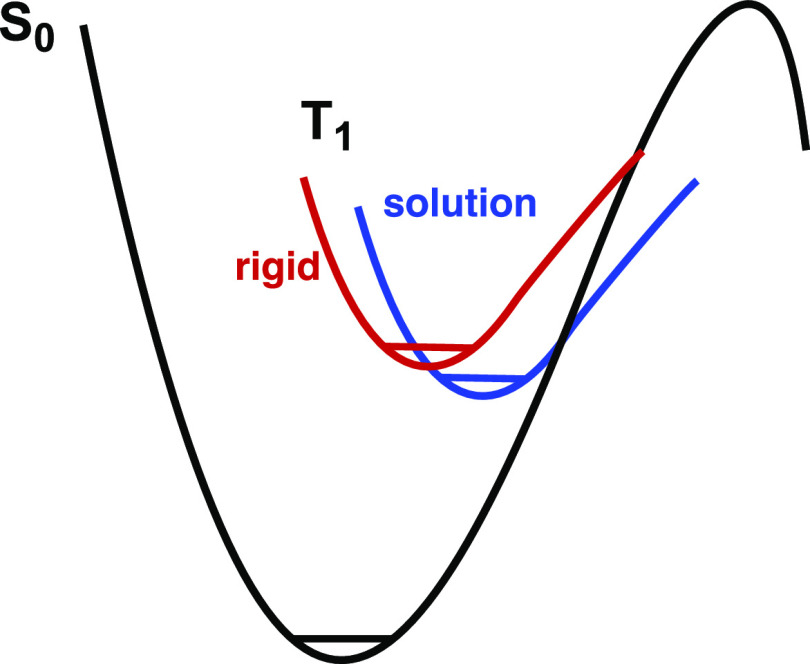
Effect of rigidification on the potential-energy surface of the excited state (T_1_) relative to the ground state (S_0_). In solution (blue curve), molecular and solvent reorientations shift the position of the potential well on the horizontal (nuclear coordinate) axis relative to the ground state. In a rigid matrix (red curve), such reorientation is diminished.

A second possibility is that nonradiative decay occurs through a thermally activated crossing between the excited-state and ground-state potential wells (the strong-coupled case) and such thermally activated crossing is facilitated through excited-state distortions (Figure 9).^70,71^ Chiefly, vertical excitation in solution is followed by vibrational relaxation to a new equilibrium geometry (Figure 9, blue) involving both the complex and the solvent orientation. The extent of distortion depends on the excited-state electron distribution. The stabilization that accompanies such distortion is partially prevented in rigid media (Figure 9, red), resulting in higher energy barriers to crossing (the weak-coupled case).^90−94^ Thus, if nonradiative decay is dominated by the barrier to potential-surface crossing, it is expected that rigidification will increase the excited-state lifetime. Furthermore, such crossing is not dependent on high-energy vibrational modes and is not expected to show a significant deuterium isotope effect (as observed). A classic example of using rigidity to impact excited-state behavior involved the immobilization of Ru(bpy)_3_^2+^ in a cellulose acetate matrix. For Ru(bpy)_3_^2+^, one of the deactivation mechanisms involves thermal access of the highly distorted ^3^MC state; rigidification renders such states thermally inaccessible.^95^ Likewise, a range of phosphors that undergo nonradiative deactivation through ^3^MC states have substantially longer lifetimes and larger photoluminescent quantum yields upon immobilization in PMMA films,^94,96−99^ or viscous media.^100^ Thus, we have investigated the effect of rigidification in a PMMA film on the photophysical characteristics of ^**Ph**^**[Ti]** and ^**Ph**^**[Ti]CuBr**.

Initially, a film of ^**Ph**^**[Ti]** in PMMA was prepared. Despite this rigidification, ^**Ph**^**[Ti]** rapidly decomposes upon irradiation, leaving a bleached region in the film at the irradiation site. This is not surprising given that this complex has a quantum yield for photodecomposition in solution of near unity. Thus, PMMA films containing ^**Ph**^**[Ti]CuBr** were investigated because of the substantially lower photodecomposition quantum yields in solution. Irradiation of this film resulted in no noticeable bleaching. However, whereas no emission is detected from ^**Ph**^**[Ti]CuBr** in solution, emission in PMMA film is observable even by eye (λ_max_ = 723 nm, τ = 4.6 μs, Figure S16). Clearly, rigidification in a matrix enhances the lifetimes and quantum yields and suggests that other means of rigidification, such as providing molecular constraint toward vibrational motions, may also enhance excited-state lifetimes.

### Investigating the Impact of Steric Bulk on Photophysics

**Figure 10:**
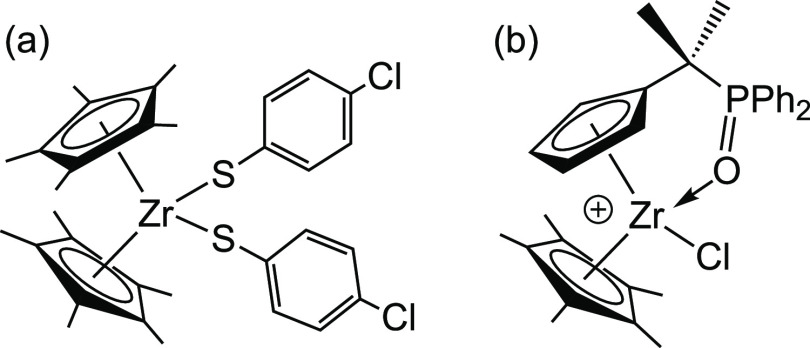
Examples of metallocenes with Cp* ligands that are emissive in RT fluid solution. For complex (a), the corresponding Cp complex is not emissive in solution,^50^ and for complex (b), the corresponding Cp complex has a lower quantum yield for emission.^54^

^**Ph**^[Cp*Ti]CuBr is emissive both at 77 K and in RT fluid solution (Figure 11). In RT solution, the emission (693 nm) is clearly visible by the eye with a quantum yield for photoluminescence, Φ_P_ = 1.3 × 10^–3^. The excitation spectra match the UV–vis spectrum, indicating that, at both RT and 77 K, emission is not due to an impurity (Figure S17). The phosphorescence lifetime is 0.18 μs (Figure S18), and both the emission intensity and lifetimes appear insensitive to whether the sample is air-saturated or purged with Ar. Furthermore, the photodecomposition quantum yield for ^**Ph**^[Cp*Ti]CuBr is 1.5 × 10^–2^ upon excitation with a 428 nm diode laser and is insensitive to whether the sample is air-saturated or purged with Ar, making this complex both more photostable (by 2 orders of magnitude) and more emissive (by 1 order of magnitude) than the previously reported emissive titanocenes, ^**Ph**^**[Ti]**, and ^**Ph**^**[Ti]AgCl**.^66^

Perhaps the simplest means to restrict molecular motions of the Cp ring is to prepare complexes with steric bulk around the Cp ring. For example, zirconocene thiolate complexes with Cp* ligands (Figure 10a) have been shown to be emissive in RT fluid solution, whereas emission from the corresponding Cp complexes was reported only in the solid-state at 77 K.^50^ Likewise, Cp* zirconocene complexes with pendant phosphine chalcogenide donors (Figure 10b) are emissive in RT solution. The photoluminescence quantum yield and lifetime for the complex shown in Figure 10b decreases by more than an order of magnitude upon replacement of Cp* with Cp; the less-restricted motion of the Cp ring was given as a possible reason.^54^ The structure of ^**Ph**^[Cp*Ti]CuBr clearly indicates the constraint imposed by the Cp* rings (*vide supra*). Chiefly, the Cp*(centroid)-Ti-Cp*(centroid) bond angle appears to be controlled by a balance of minimizing methyl–methyl and methyl-phenyl nonbonding interactions, perhaps constraining centroid-Ti-centroid bending vibrations. Substitution at the Cp ring is also expected to increase the barrier to rotation. For example, although the rotational barrier for Cp_2_TiCl_2_ is estimated as 1 kcal/mol,^101^ the addition of two SiMe_3_ substituents on each Cp ring increases that to 8.9 kcal/mol as measured using coalescence of the ^1^H NMR spectrum.^102^ To the best of our knowledge, the rotational barrier for Cp*_2_TiCl_2_ or related titanocenes has not been measured. However, the ^1^H NMR spectrum for Cp*_2_TiCl_2_ remains a sharp singlet down to −50 °C.^86^ Regardless, the photophysical properties of ^**Ph**^[Cp*Ti]CuBr were investigated as a test of whether such steric bulk might provide the necessary restriction to excited-state reorganization. The bulk of the methyl substituents may also serve to minimize the excited-state solvent reorganization. The coordination of CuBr is to improve photostability (*vide supra*).

**Figure 11:**
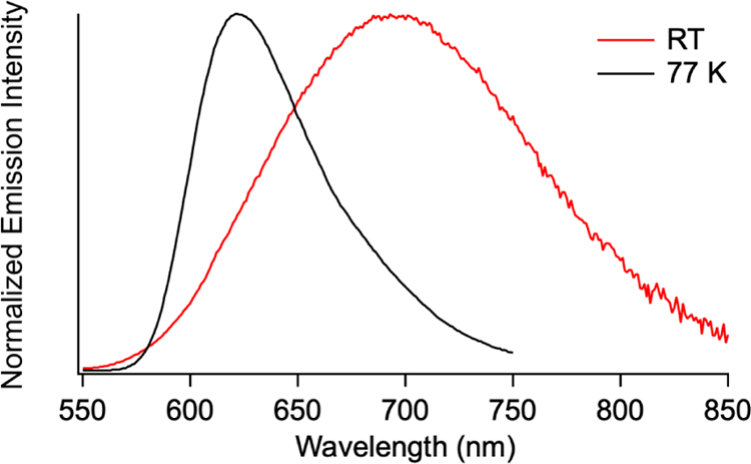
Overlay of normalized emission spectra of ^**Ph**^[Cp*Ti]CuBr at 77 K in 2-methyltetrahydrofuran (λ_ex_ = 382 nm) and in RT THF solution (λ_ex_ = 430 nm).

**Figure 12:**
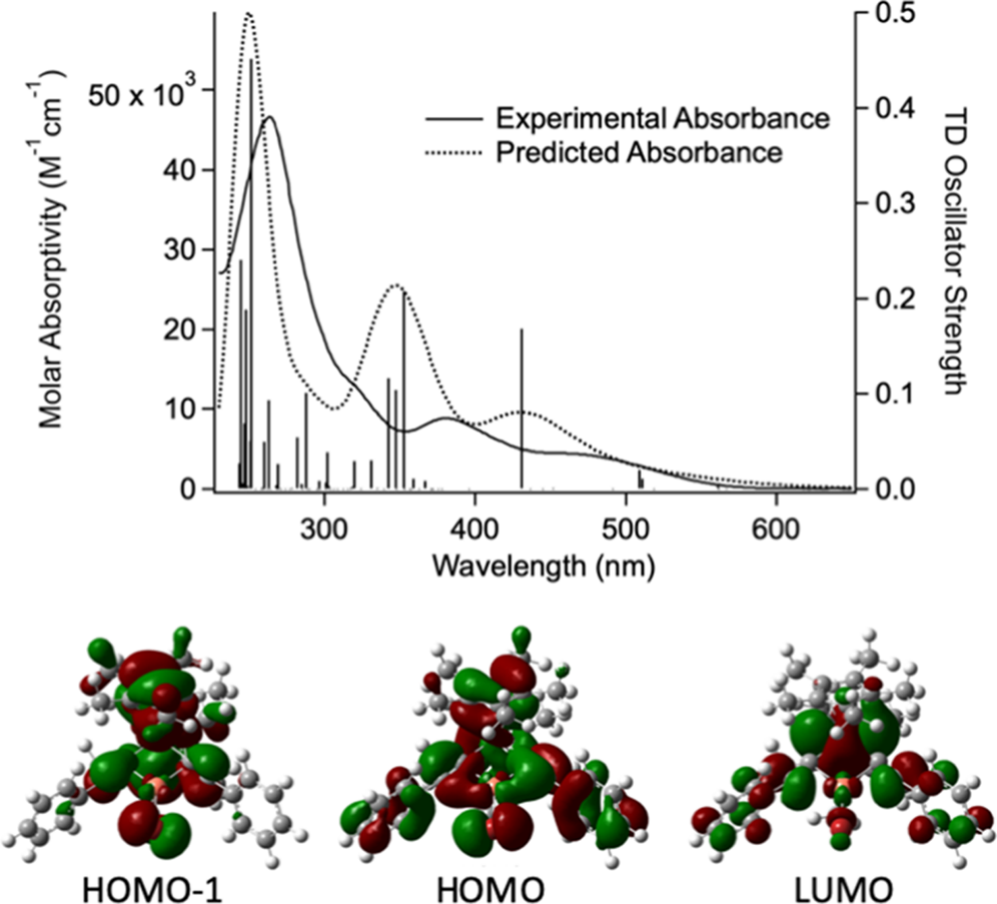
Overlay of UV–vis spectrum (THF) and TDDFT (MN15/LANL2DZ//B3LYP/6-311+G(d))-predicted vertical transitions for ^**Ph**^[Cp*Ti]CuBr, as well as the predicted UV–vis spectrum (top) and the HOMO – 1, HOMO, and LUMO for ^**Ph**^[Cp*Ti]CuBr (bottom).

Computational modeling of ^**Ph**^[Cp*Ti]CuBr was performed using the MN15/LANL2DZ//B3LYP/6-311+G(d) (TDDFT//DFT) model, shown to accurately predict the spectral properties of the ^**R**^**[Ti]MX** complexes.^66^ The predicted UV–vis spectrum is in good agreement with the experimental UV–vis spectrum (Figure 12) but is blue-shifted by approximately 2000 cm^–1^. One possible explanation for the discrepancy is that the phenyl substituents for ^**Ph**^[Cp*Ti]CuBr are perpendicular to the Ti-alkyne-CuBr plane in the DFT optimized geometry, whereas the phenyl rings are tilted 30° or less from that plane in the crystal structure. It may be that the solution geometry is closer to that of the crystal structure than the DFT optimization. Though the lowest-energy singlet with significant oscillator strength (431 nm) is dominated by a HOMO to LUMO transition, the lowest-energy singlet and triplet are dominated by HOMO – 1 to LUMO and population analysis shows that these transitions are dominated by a Cp* to Ti LMCT (Charts S3 and S4 and Table S2). It is this lowest-energy triplet that will dominate the photophysics. This change in lowest-energy excited-state character for ^**Ph**^[Cp*Ti]CuBr vs ^**Ph**^**[Ti]CuBr** is precedented in titanocenes. For example, the lowest-energy excited state for Cp_2_TiI_2_ has been assigned to I-to-Ti LMCT, but as Cp*-to-Ti LMCT in the analogous Cp*_2_TiI_2_ complex.^103^

Despite Cp* being bulkier than Cp, it is not clear that steric restriction caused by this additional bulk is the reason for the remarkable RT solution photophysics of ^**Ph**^[Cp*Ti]CuBr compared to ^**Ph**^**[Ti]CuBr** (which is nonemissive in RT solution). Other possible explanations include: (1) The nature of the excited state has changed to be dominated by Cp* to Ti LMCT with no phenylethynyl-to-Ti LMCT. (2) Replacement of the H atoms on the Cp ring with CH_3_ groups precludes those high-energy C–H vibrations from being acceptor modes for the energy in the electronic excited state. (3) The lowest-energy triplet (as measured by 77 K emission) is higher in energy for ^**Ph**^[Cp*Ti]CuBr (622 nm) compared to ^**Ph**^**[Ti]CuBr** (715 nm) and this may lead to a higher barrier for surface crossing for the former (*vide supra*). (4) Excited-state solvent reorganization may be diminished by the replacement of Cp by Cp*. For a related set of titanocenes of the type ^R^Cp_2_Ti(C_2_Fc)_2_, replacement of Cp by Cp* decreased the dependence of the absorption energy on solvent.^104^

## Conclusions and Outlook

The observation that ^**OBET**^**[Ti]** is significantly less photostable than ^**Ph**^**[Ti]CuX** complexes provides further evidence that the structural constraint of the arylalkynes is not sufficient to explain the relatively high photostability of the ^**R**^**[Ti]CuX** complexes. The current results are thus consistent with the previous hypothesis that the dominant role that CuX binding plays in increasing the photostability of the arylethynyltitanocenes is the lowering of the excited-state energy, rendering the transition state for decomposition less accessible.

The lack of a deuterium isotope effect on the photophysics of ^**Ph**^**[Ti]** and ^**Ph**^**[Ti]CuX** led to an investigation of the role of rigidification. For ^**Ph**^**[Ti]CuBr**, rigidification in a PMMA film leads to RT emission with a 4.6 μs lifetime, whereas emission is absent in RT fluid solution. This work adds to the growing body of literature suggesting that rigidification of d^0^ metallocenes may significantly enhance emission intensity and lifetimes in RT fluid solution,^51,53−55^ suggesting a strong-coupled mechanism for nonradiative decay. Such a mechanism would also be likely to demonstrate a dependence of the observed emission intensity (in RT fluid solution) on the energy of the excited state, inasmuch as lowering the excited-state energy would also lower the activation barrier for crossing between potential-energy surfaces for partially nested potential wells (Figure 9). As such, this result is consistent with the previously reported lack of emission for ^**DMA**^**[Ti]** and ^**TPA**^**[Ti]** in RT fluid solution, considering that the emission wavelengths for those complexes (measured at 77 K) are significantly red-shifted (672 and 642 nm, respectively) relative to that of ^**Ph**^**[Ti]** (575 nm).^66^ Furthermore, with the exception of ^**Ph**^**[Ti]AgCl**, none of the ^**R**^**[Ti]MX** complexes are emissive in RT solution. Once again, the emission maxima (77 K) of the ^**R**^**[Ti]MX** complexes (659–767 nm) are red-shifted relative to that of the only MX coordinated complex that is emissive in room-temperature solution ^**Ph**^**[Ti]AgCl** (578 nm).^66^

Of particular interest is the finding that ^**Ph**^[Cp*Ti]CuBr is emissive in RT fluid solution (Φ_P_ = 1.3 × 10^–3^, τ = 0.18 μs). Within this context, it is noteworthy that most metallocenes reported to be emissive in RT fluid solution are also Cp* derivatives.^46−50,54,55^ Work is ongoing in our laboratory to investigate the role that Cp* plays in the photophysical properties, and to investigate the possible use of ^**Ph**^[Cp*Ti]CuBr and related complexes as photocatalysts.

## Supporting Information Available

NMR spectra for complexes prepared herein; crystallographic data, packing arrangement, and several different orientations for the solid-state structure of ^**Ph**^**[Cp*Ti]CuBr**; representative NMR spectra for photodecomposition quantum yield measurements for ^**OBET**^**[Ti]**; emission spectra, excitation spectra, and luminescence decay curves; orbital contributions for lowest-energy triplet and singlets and frontier orbital images for ^**OBET**^**[Ti]** and ^**Ph**^**[Cp*Ti]CuBr**; and population analysis for lowest-energy triplet and singlets for ^**Ph**^**[Cp*Ti]CuBr** (PDF)
Cartesian coordinates for optimized structures of ^**OBET**^**[Ti]** using MN15/LANL2DZ and ^**Ph**^**[Cp*Ti]CuBr** using B3LYP/6-311+G(d) (XYZ)

## Supplementary Material

ic3c02712_si_001.pdf

ic3c02712_si_002.xyz
