# Supplementary material for: Design Strategies for Luminescent Titanocenes: Improving the Photoluminescence and Photostability of Arylethynyltitanocenes
Source: Inorg Chem. 2023 Oct 13;62(43):17870–82. doi: 10.1021/acs.inorgchem.3c02712 (PMC10618925; doi:10.1021/acs.inorgchem.3c02712)
Supplement: Supplementary file 1 — ic3c02712_si_001.pdf [file ic3c02712_si_001.pdf]

Supporting Information

Design Strategies for Luminescent Titanocenes: Improving the Photoluminescence and Photostability of Arylethynyltitanocenes

Matilda Barker,[a] Thomas J. Whittemore,[a] Henry C. London,[a] Jack M. Sledesky,[a] Elizabeth A. Harris,[a] Tiffany M. Smith Pellizzeri,[b] Colin D. McMillen,[c] Paul S. Wagenknecht*,[a]

[a] Department of Chemistry, Furman University, Greenville, SC 29609. [b] Department of Chemistry and Biochemistry, Eastern Illinois University, Charleston, IL 61920. [c] Department of Chemistry, Clemson University, Clemson, SC 29634.

* Email address of the corresponding author:

Paul S. Wagenknecht: paul.wagenknecht@furman.edu

Table of Contents **Page**

^{1}H NMR spectra (Figures S1-S5)	1-5
Data and diagrams for solid state structure of ^{Ph}**[Cp*Ti]CuBr** (Table S1, Figs S6-S8)	6-9
Orbital Contributions to lowest-energy transitions for ^{OBET}**[Ti]** (Charts S1, S2)	10-13
Emission and excitation spectra, luminescence decay fit for ^{OBET}**[Ti]** (Figs S9-S10)	14
^{1}H NMR spectra for determination of Φ_{decomp} for ^{OBET}**[Ti]** (Fig S11)	15
^{1}H NMR spectrum of decomposition products for ^{OBET}**[Ti]** in C_6H_6 (Fig S12)	16
Luminescence decay fits for protio and deuterio ^{Ph}**[Ti]** and ^{Ph}**[Ti]CuX** (Figs S13 – S15)	17
Emission spectrum and luminescence decay fit for PMMA film of ^{Ph}**[Ti]CuBr** (Fig S16)	18
Excitation spectra for ^{Ph}**[Cp*Ti]CuBr** (Fig S17)	18
Luminescence decay fit for ^{Ph}**[Cp*Ti]CuBr** (Fig S18)	18
Orbital Contributions to lowest-energy transitions for ^{Ph}**[Cp*Ti]CuBr** (Charts S3, S4)	19-29
Mulliken population analysis for ^{Ph}**[Cp*Ti]CuBr** (Table S2)	30

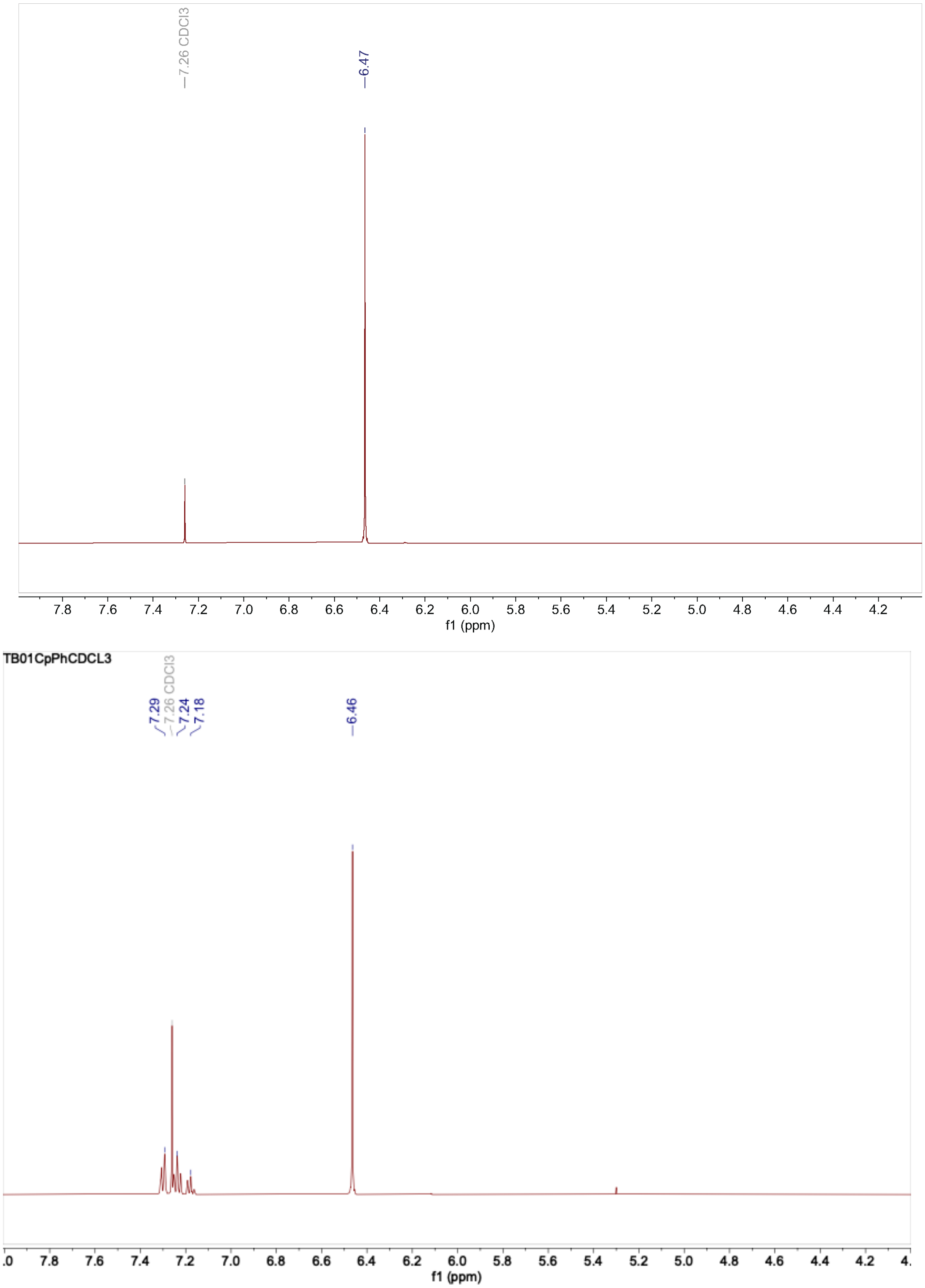

Figure S1: ^{1}H NMR spectra of $Cp_2Ti(C_2Ph)_2$ (500 MHz, top, $CDCl_3$) and $Cp_2Ti(C_2Ph)_2$-d_{10} (500 MHz, bottom, $CDCl_3$).

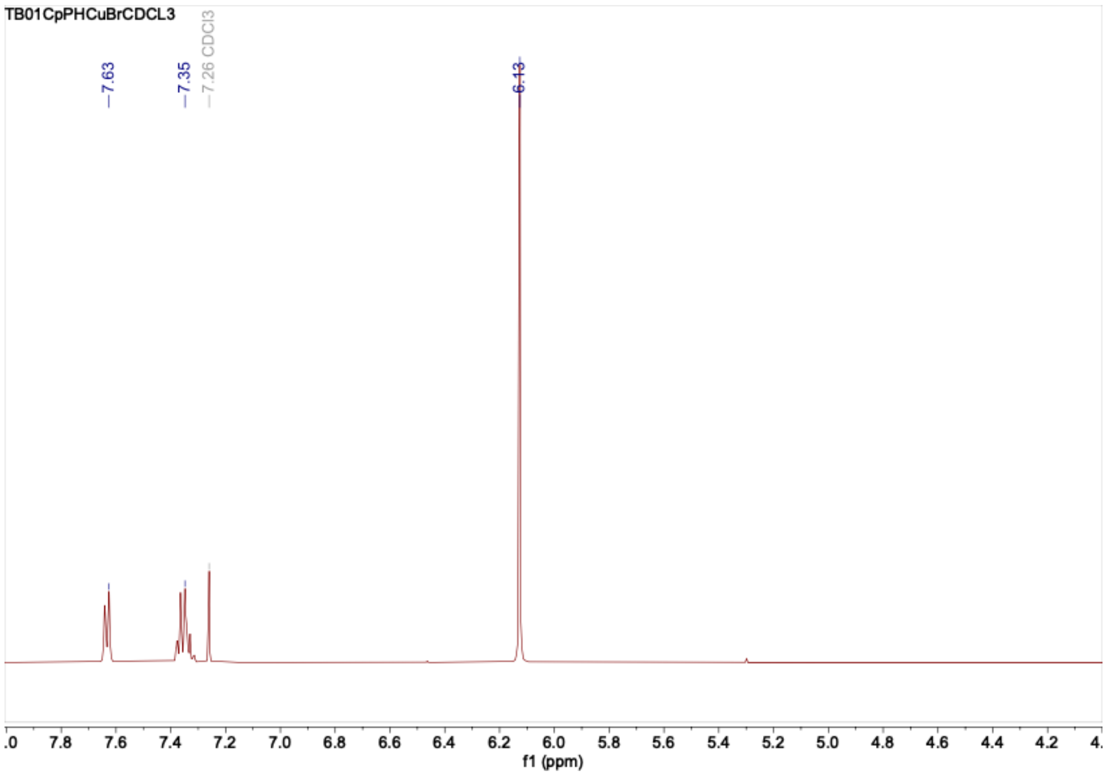

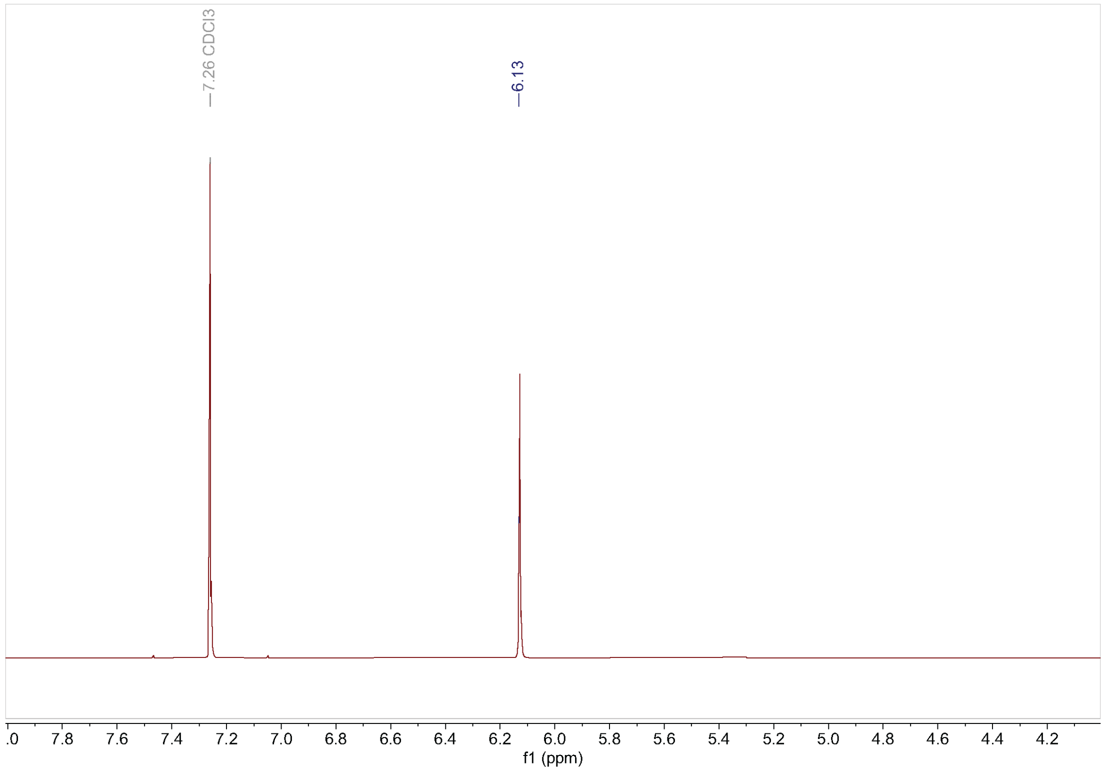

Figure S2: ^{1}H NMR spectra of $Cp_2Ti(C_2Ph)_2CuBr$ (500 MHz, top, $CDCl_3$) and $Cp_2Ti(C_2Ph)_2CuBr$-*d_{10}* (500 MHz, bottom, $CDCl_3$).

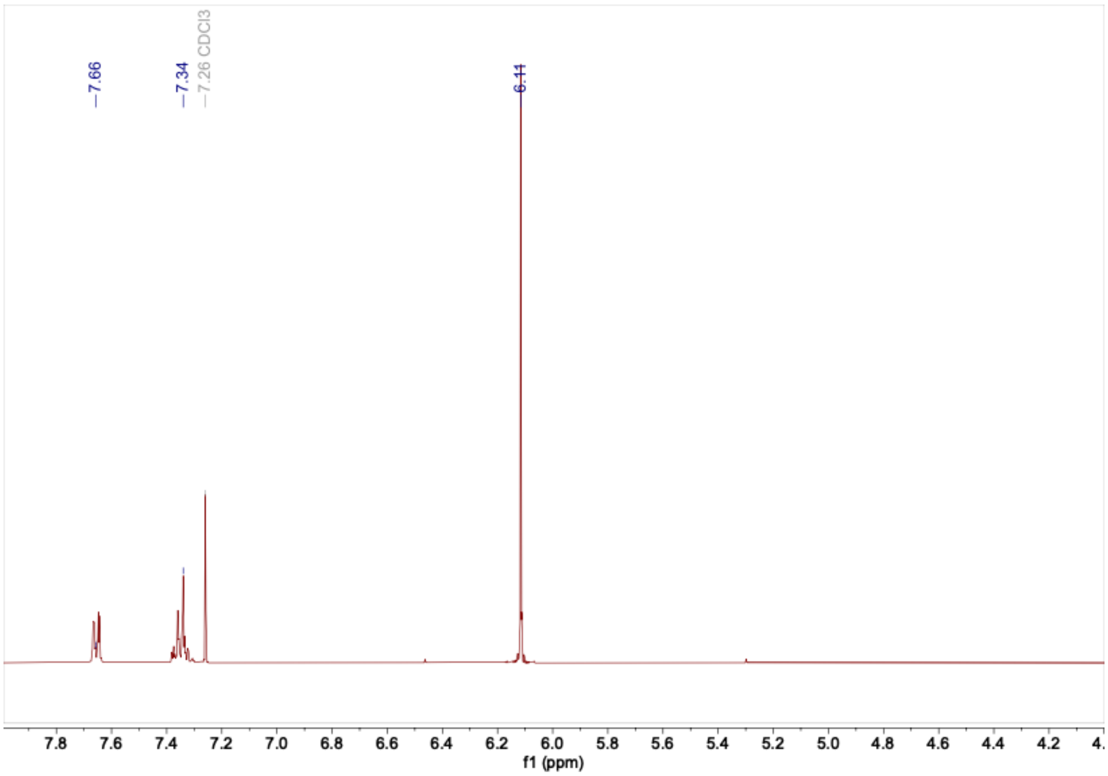

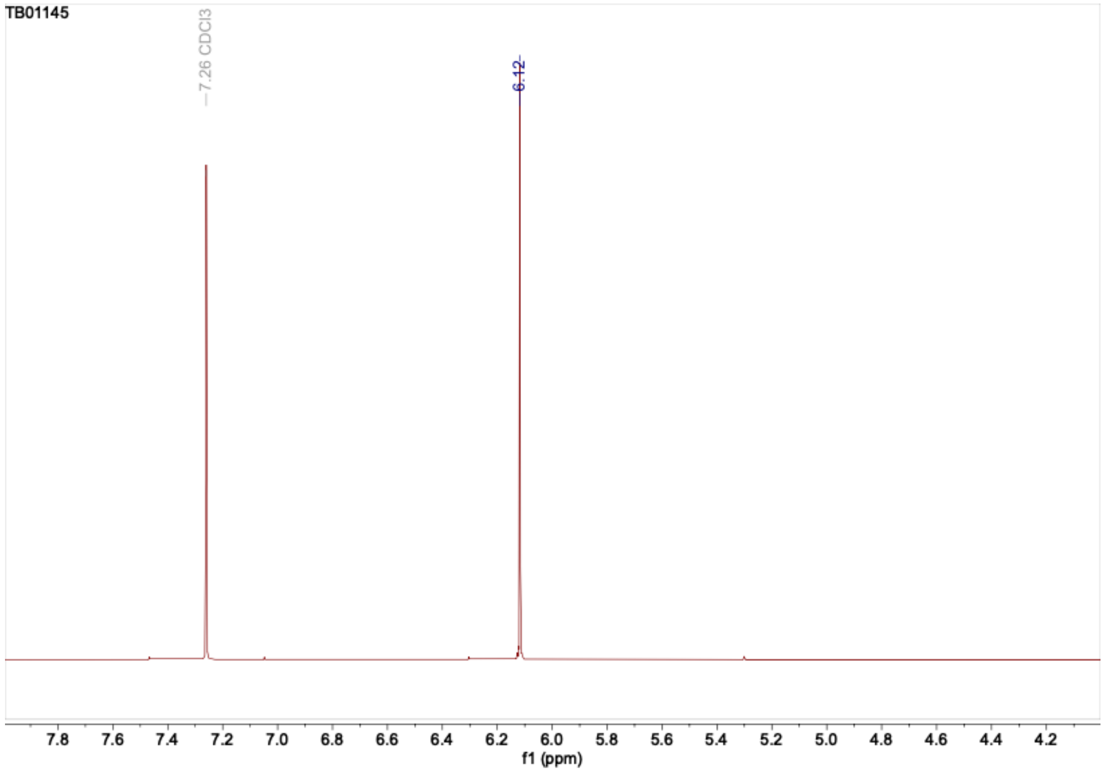

Figure S3: ^{1}H NMR spectra of $Cp_2Ti(C_2Ph)_2CuCl$ (500 MHz, top, $CDCl_3$) and $Cp_2Ti(C_2Ph)_2CuCl$-d_{10} (500 MHz, bottom, $CDCl_3$).

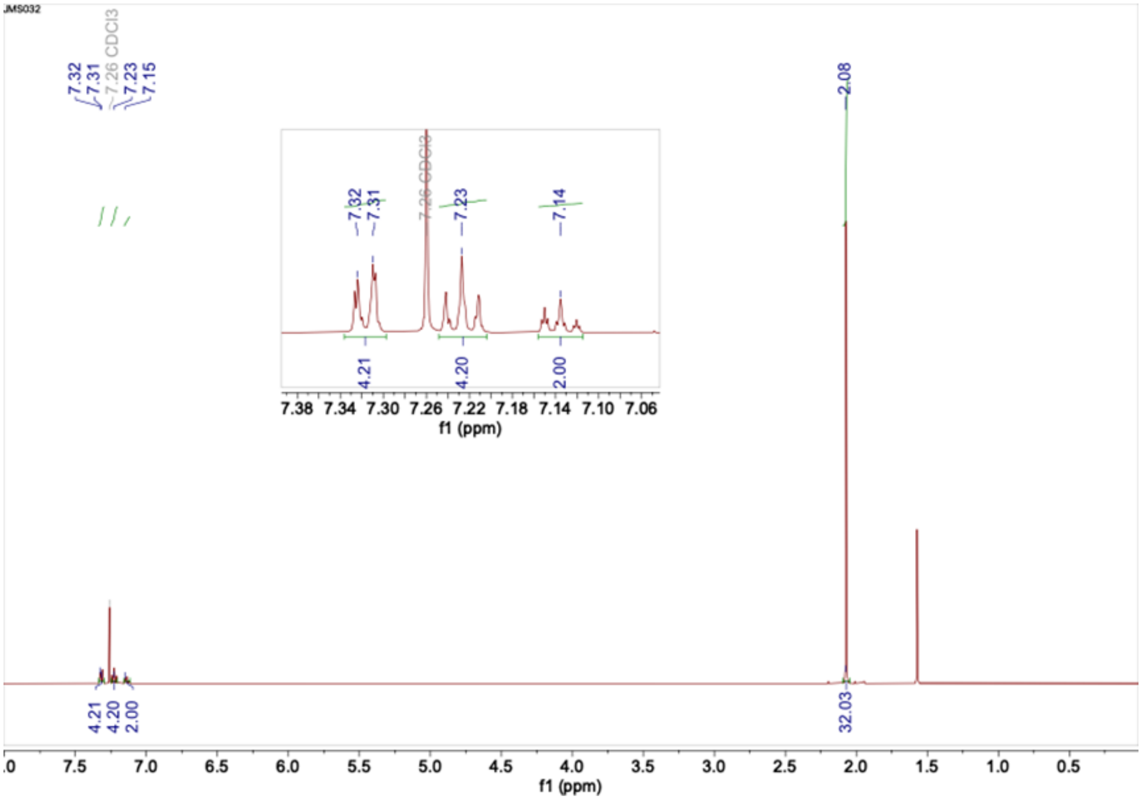

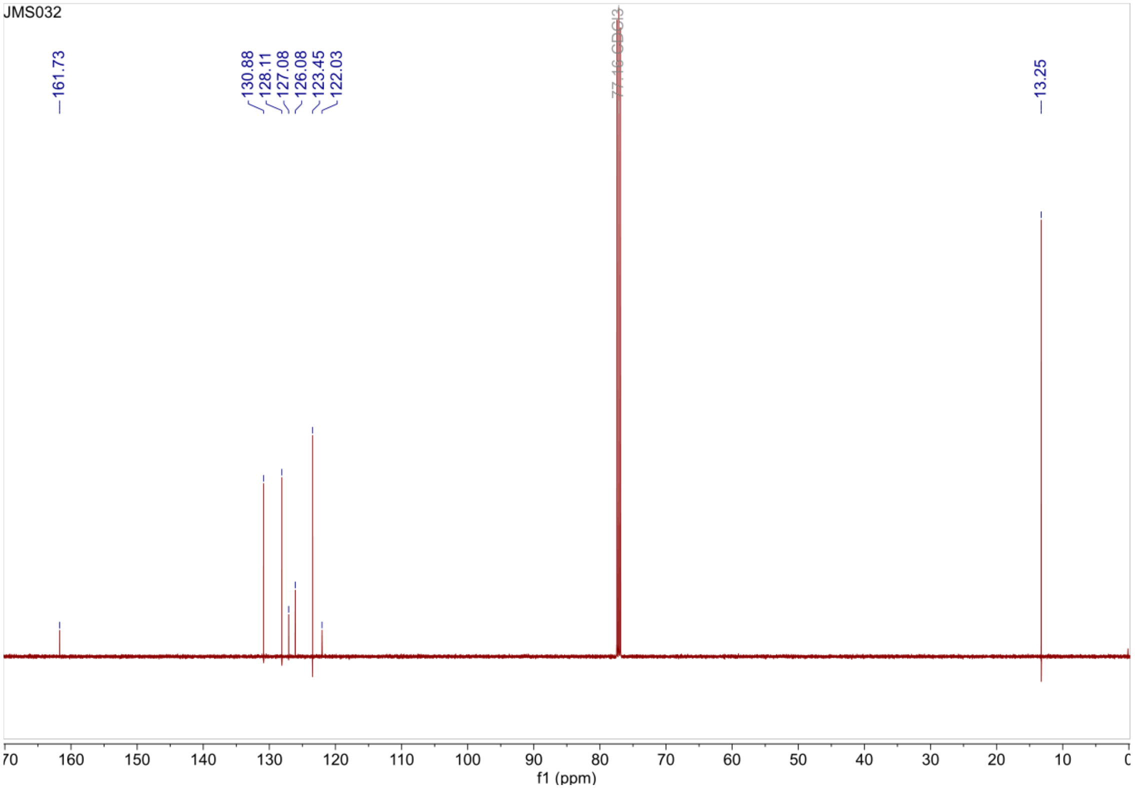

Figure S4: ^{1}H NMR spectrum (500 MHz, top, $CDCl_3$) and ^{13}C NMR spectrum (125 MHz, bottom, $CDCl_3$) of $Cp^*{}_2Ti(C_2Ph)_2$.

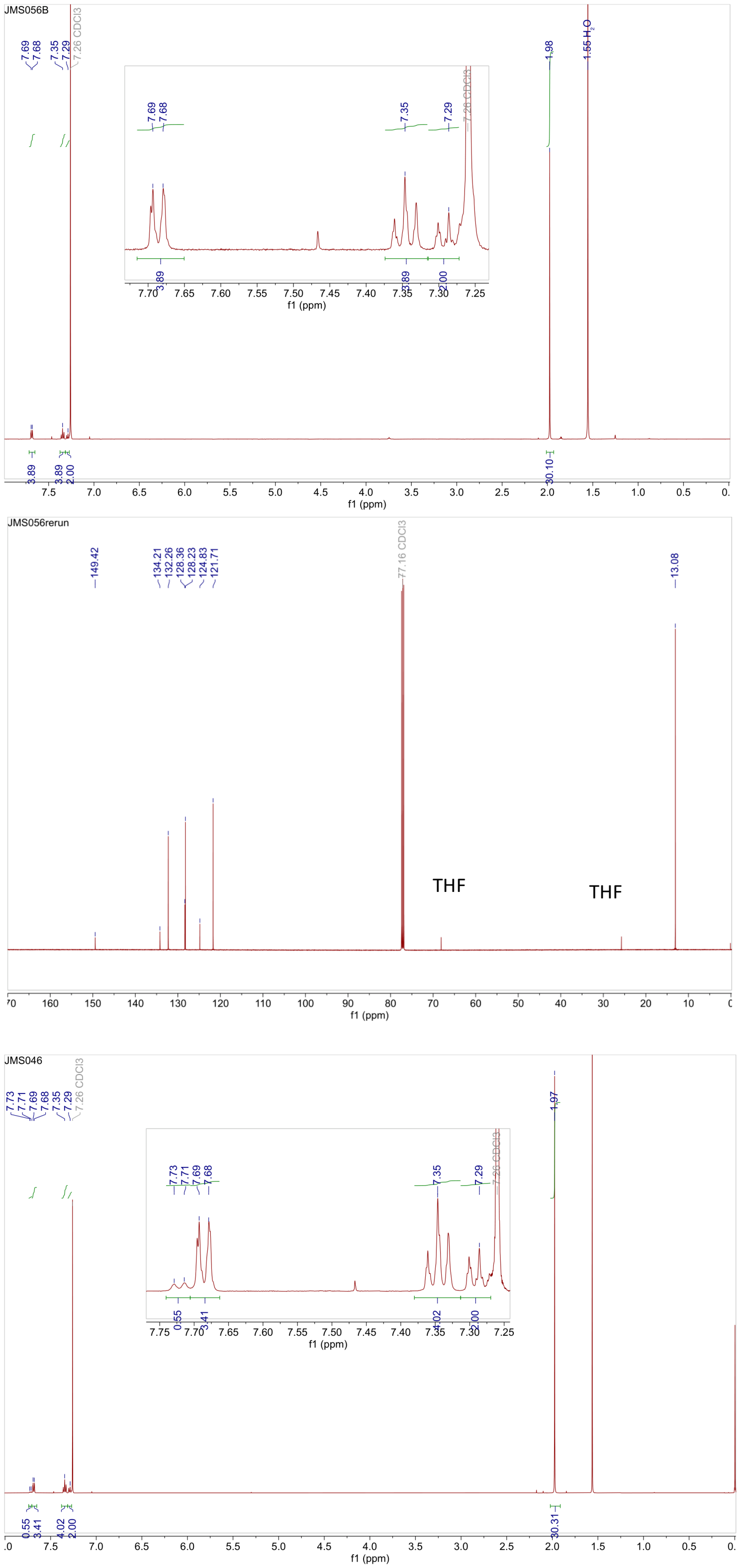

Figure S5: ^{1}H NMR spectrum (500 MHz, top, $CDCl_3$) and ^{13}C NMR spectrum (125 MHz, middle, $CDCl_3$) of $Cp^*_2Ti(C_2Ph)_2CuBr$ using the recrystallization purification method. ^{1}H NMR spectrum (500 MHz, bottom, $CDCl_3$) of $Cp^*_2Ti(C_2Ph)_2CuBr$ using column chromatography for purification. Note the additional small doublet at 7.72 ppm.

Table S1. Crystallographic data.

	**Ph[Cp*Ti]CuBr**
empirical formula	$C_{36}H_{40}BrCuTi$
formula wt. (g/mol)	664.03
crystal system	tetragonal
space group, *Z*	$P4_32_12$, 4
temperature (K)	100(2)
crystal size (mm)	0.10, 0.10, 0.03
a (Å)	9.9452(2)
c (Å)	30.6546(11)
volume (Å^3)	3031.95(16)
D_{calc} (g/cm^3)	1.455
abs. coeff. (mm^{-1})	4.806
F(000)	1368
Θ range for data	4.67-66.81
reflections coll.	12961
data/restr./param.	2682/492/307
abs. struct. param. (Flack)	0.000(18)
R(int)	0.0851
R1, wR2 [$I > 2\sigma(I)$]	0.0544, 0.1252
R1, wR2 (all data)	0.0894, 0.1453
goodness-of-fit on F^2	1.012
larg. diff. peak, hole (eÅ^{-3})	0.341, -0.644
CCDC Deposition No.	2286856

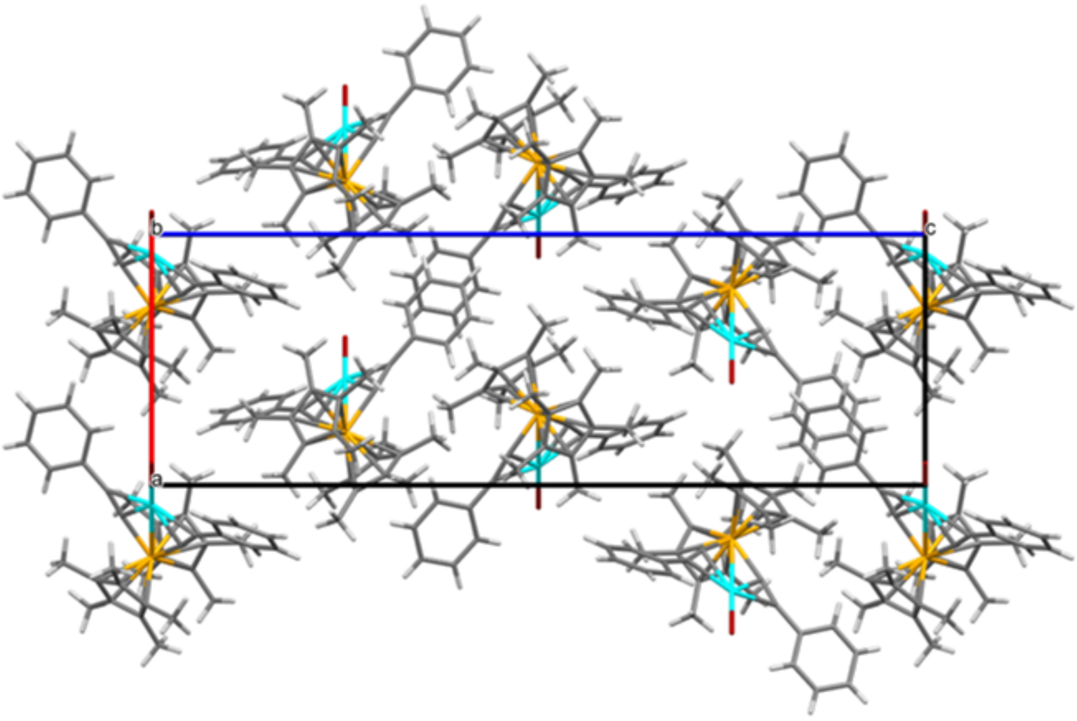

Figure S6. Packing of ^{Ph}**[Cp*Ti]CuBr** complexes viewed along the *b*-axis. For clarity, only atoms of the majority-occupied disordered arrangement are shown.

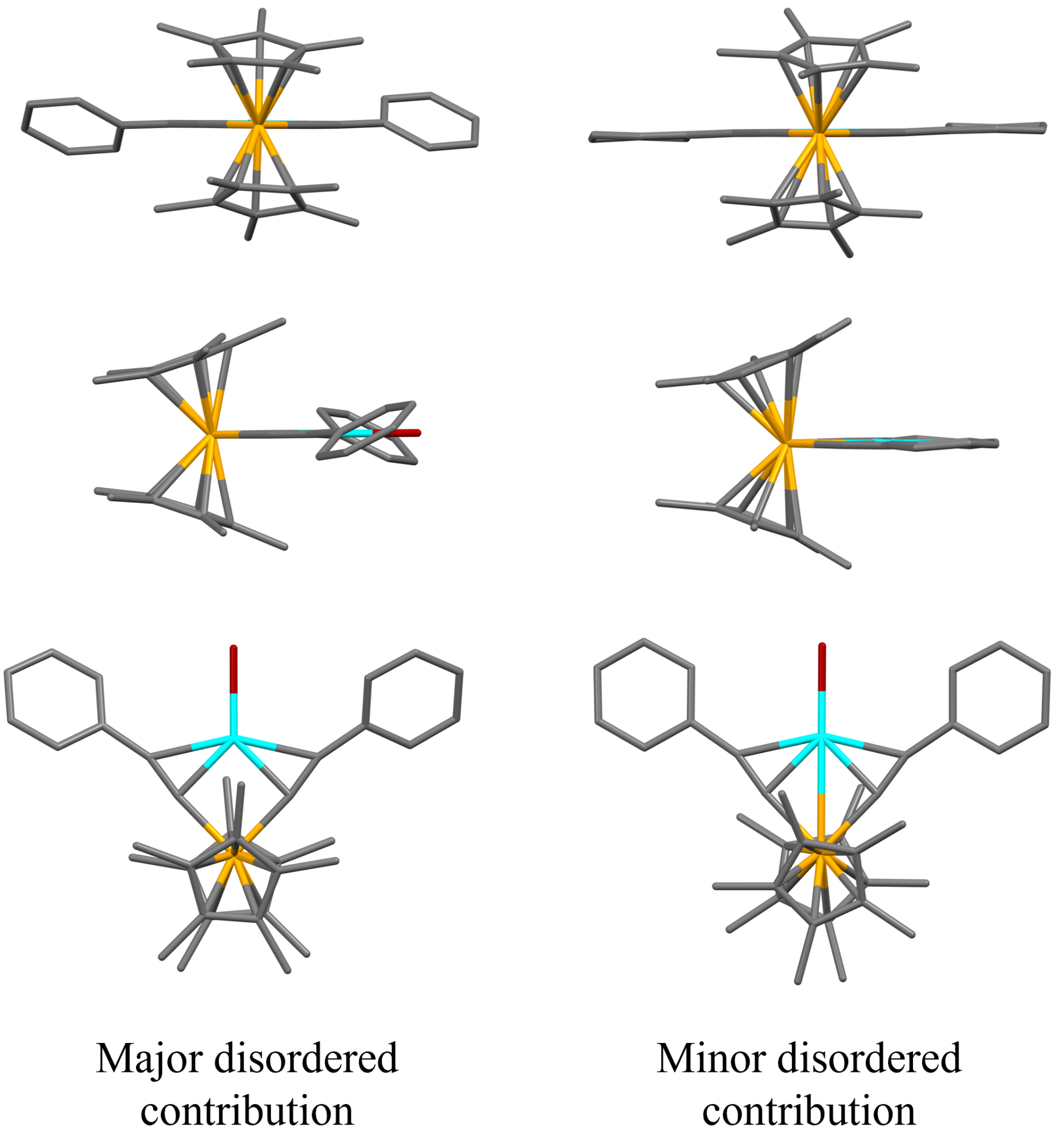

Figure S7. Tube diagram showings the major (left) and minor (right) disordered arrangements in **[Ph][Cp*Ti]CuBr**. Hydrogen atoms are omitted for clarity.

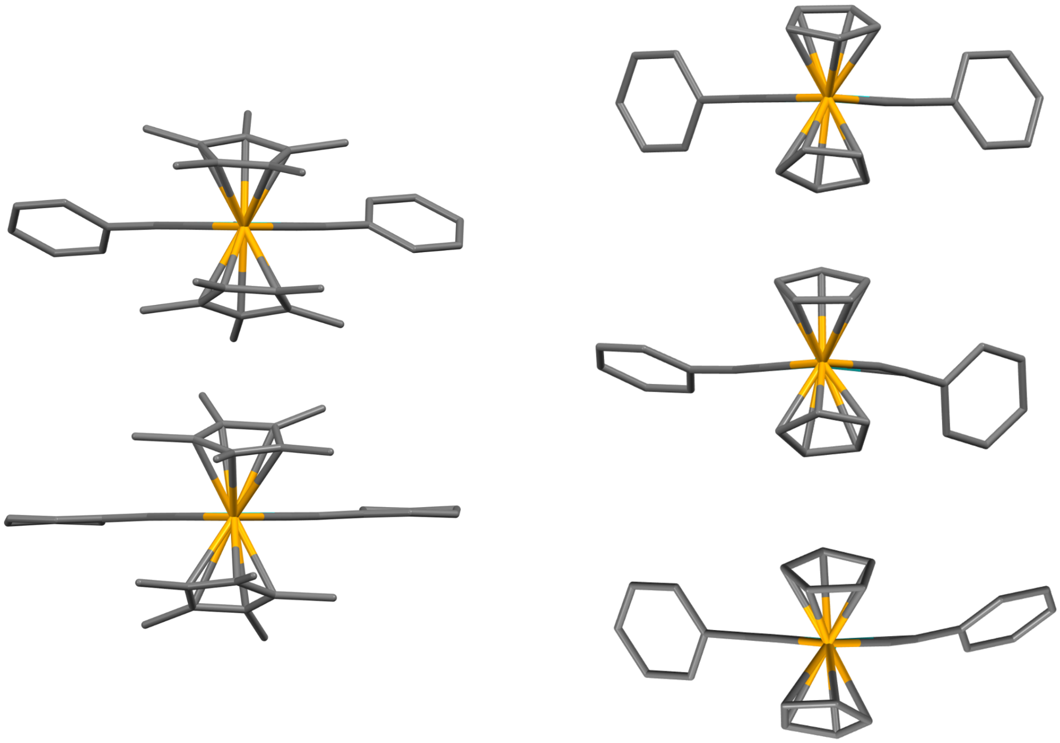

^{Ph}**[Cp*Ti]CuBr** ^{Ph}**[CpTi]CuBr**

Figure S8. Tube diagrams viewed along the Ti-alkyne-Cu-Br plane in the structures of ^{Ph}**[Cp*Ti]CuBr** (left, showing both disordered contributions) and ^{Ph}**[CpTi]CuBr** (right, showing all three unique complexes in the structure, data from reference 66). Hydrogen atoms are omitted for clarity.

Chart S1: Orbital contributions to the singlet transitions and lowest-energy triplet transition for Cp_2Ti(OBET) using MN15/LANL2DZ//MN15/LANL2DZ.

Excited State 1: Triplet-A 2.0488 eV 605.15 nm f=0.0000 <S**2>=2.000
95 ->100 0.19212
97 ->100 0.25981
99 ->100 0.62353

Excited State 2: Singlet-A 2.1822 eV 568.15 nm f=0.0015 <S**2>=0.000
95 ->100 0.12882
97 ->100 0.22967
99 ->100 0.65074

Excited State 5: Singlet-A 2.5652 eV 483.34 nm f=0.0004 <S**2>=0.000
93 ->100 -0.13750
98 ->100 0.67600

Excited State 8: Singlet-A 3.1240 eV 396.87 nm f=0.0380 <S**2>=0.000
84 ->100 -0.10150
92 ->100 -0.28110
96 ->100 0.62206

Excited State 12: Singlet-A 3.3657 eV 368.38 nm f=0.0029 <S**2>=0.000
87 ->100 -0.13815
95 ->100 0.44124
97 ->100 0.46351
99 ->100 -0.24299

Excited State 15: Singlet-A 3.5457 eV 349.68 nm f=0.0463 <S**2>=0.000
92 ->100 0.17937
97 ->103 -0.12665
99 ->101 0.63716
99 ->103 -0.11624

Excited State 18: Singlet-A 3.6151 eV 342.96 nm f=0.0020 <S**2>=0.000
85 ->100 0.12150
90 ->100 0.40667
91 ->100 0.31211
93 ->100 0.16581
94 ->100 -0.36134
99 ->102 0.21252

Excited State 19: Singlet-A 3.6626 eV 338.52 nm f=0.0182 <S**2>=0.000

84 ->100 0.19010
92 ->100 0.53924
96 ->100 0.28700
98 ->102 0.18814
99 ->101 -0.14428

Excited State 20: Singlet-A 3.7701 eV 328.86 nm f=0.1330 <S**2>=0.000
90 ->100 -0.21551
93 ->100 -0.12741
97 ->102 0.15489
99 ->102 0.60081

Excited State 22: Singlet-A 3.7912 eV 327.03 nm f=0.0098 <S**2>=0.000
88 ->100 -0.25571
90 ->100 0.17969
91 ->100 -0.38846
93 ->100 0.41333
94 ->100 0.13383
98 ->100 0.13681
99 ->102 0.12173

Excited State 24: Singlet-A 3.8667 eV 320.65 nm f=0.0145 <S**2>=0.000
91 ->102 0.12734
92 ->100 -0.10116
93 ->102 -0.15784
95 ->101 0.10502
95 ->103 -0.16656
97 ->101 0.12500
97 ->103 -0.13242
98 ->102 0.43417
99 ->103 -0.35815
99 ->107 -0.11044

Excited State 25: Singlet-A 3.9357 eV 315.02 nm f=0.0008 <S**2>=0.000
87 ->100 0.21436
95 ->100 -0.45245
97 ->100 0.46794

Chart S2. Frontier orbitals corresponding to the lowest energy triplet and singlets for Cp_2Ti(OBET) using MN15/LANL2DZ.[a]

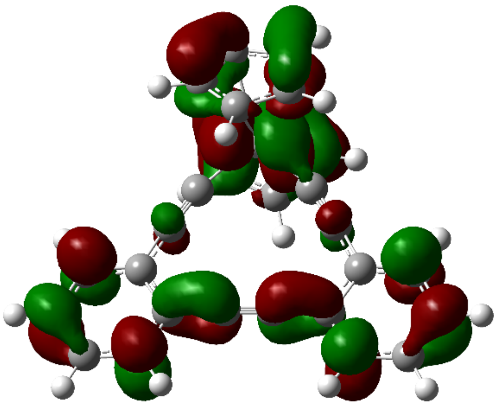	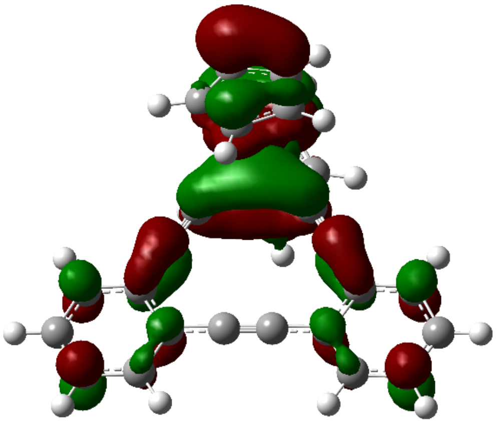
LUMO+3 (MO=103, Energy=-0.01884)	LUMO+2 (MO=102, Energy=-0.03498)
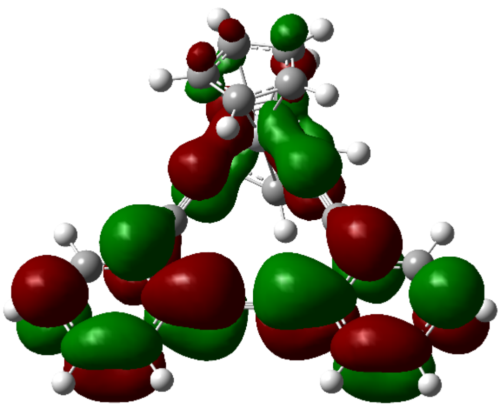	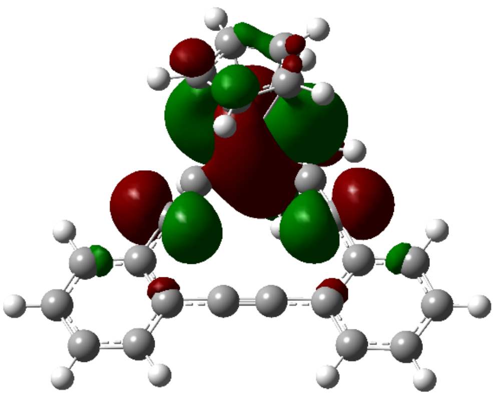
LUMO+1 (MO=101, Energy=-0.04263)	LUMO (MO=100, Energy=-0.08044)
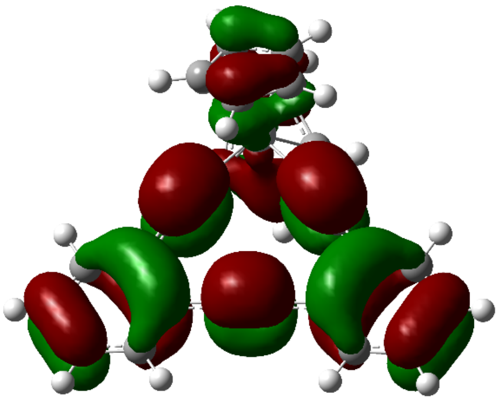	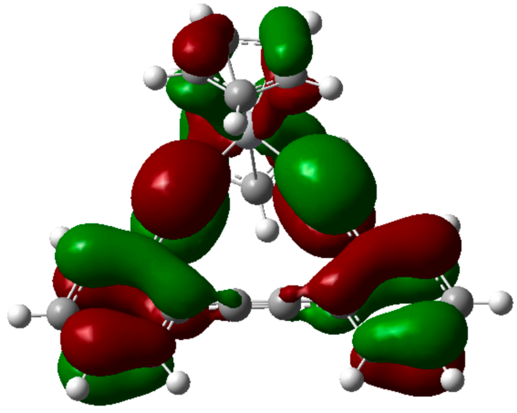
HOMO (MO=99, Energy=-0.23494)	HOMO-1 (MO=98, Energy=-0.25295)

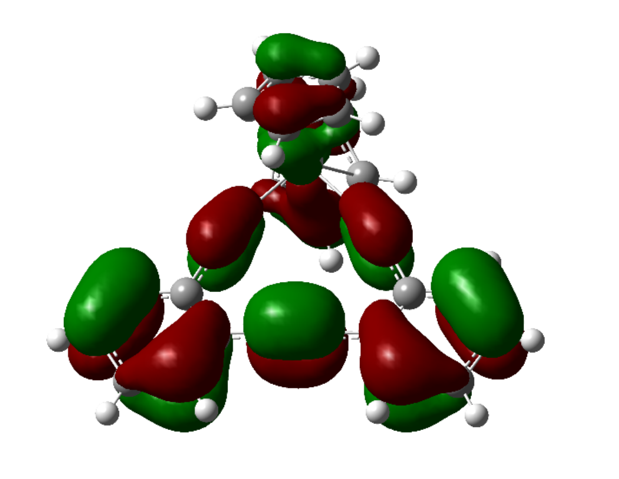

HOMO-2 (MO=97, Energy=-0.26587)

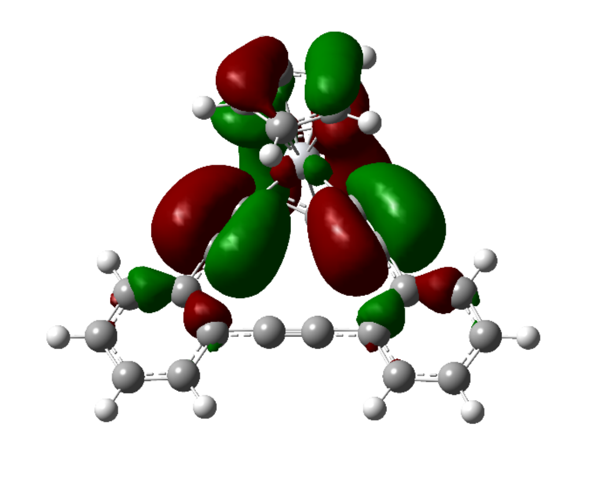

HOMO-3 (MO=96, Energy=-0.27511)

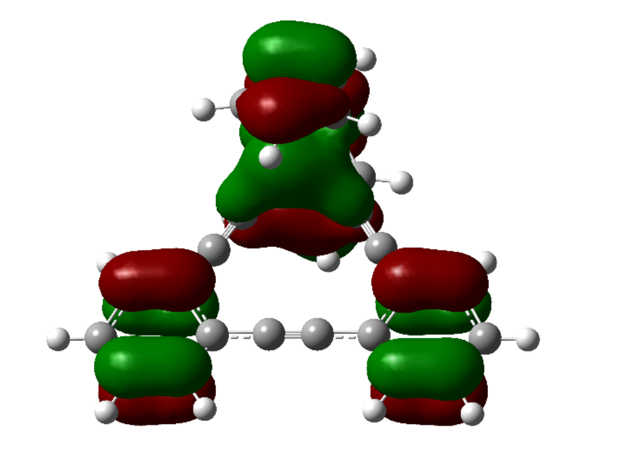

HOMO-4 (MO=95, Energy=-0.30144)

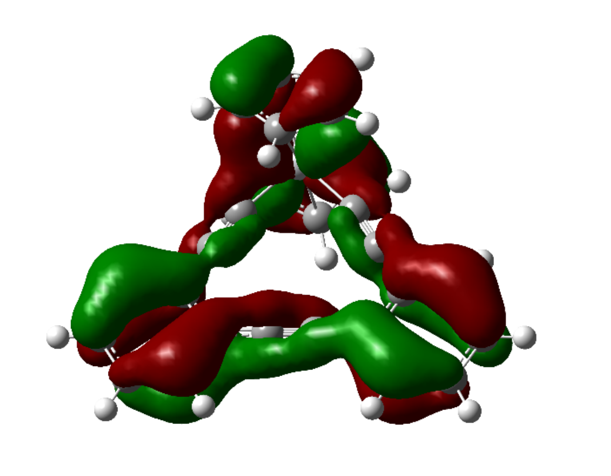

HOMO-6 (MO=93, Energy=-0.30904)

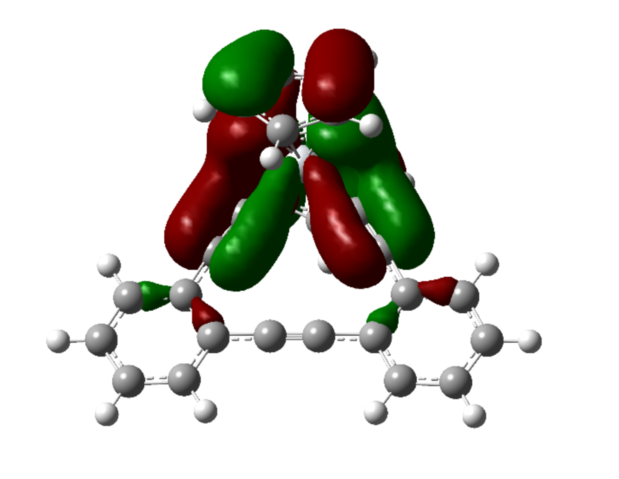

HOMO-7 (MO=92, Energy=-0.31025)

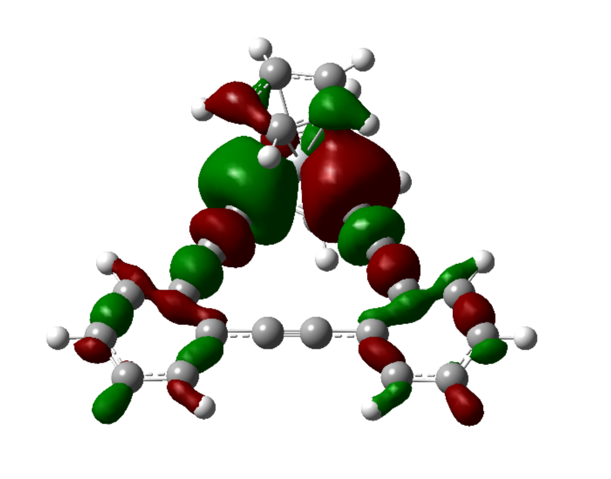

HOMO-15 (MO=84, Energy=-0.37820)

[a]A default isovalue of 0.02 is used for all surfaces.

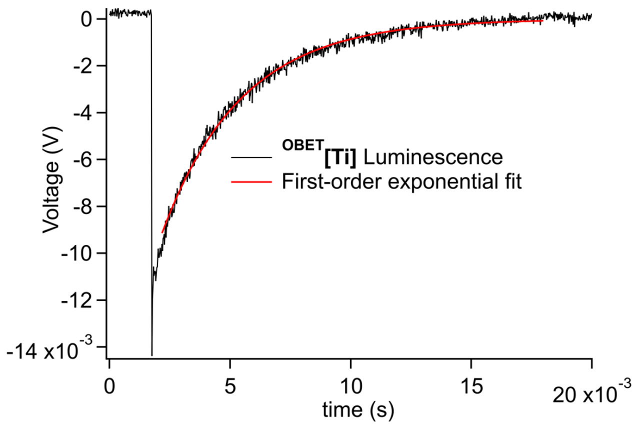

Figure S9: Luminescence decay curve for Cp_2Ti(OBET) collected in a 77 K matrix of 2-methyltetrahydrofuran overlaid with a first-order exponential fit. λ_{ex} = 367 nm, λ_{em} = 600 nm.

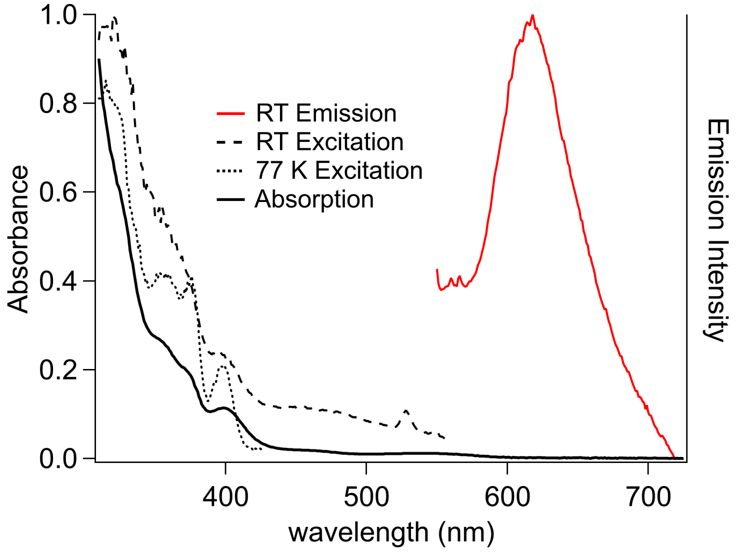

Figure S10: Emission spectrum of Cp_2Ti(OBET) collected in a room-temperature fluid solution of THF (λ_{ex}=398 nm, λ_{max}=618 nm) overlaid with the excitation spectrum (λ_{em}=625 nm) and the UV-Vis absorption spectrum.

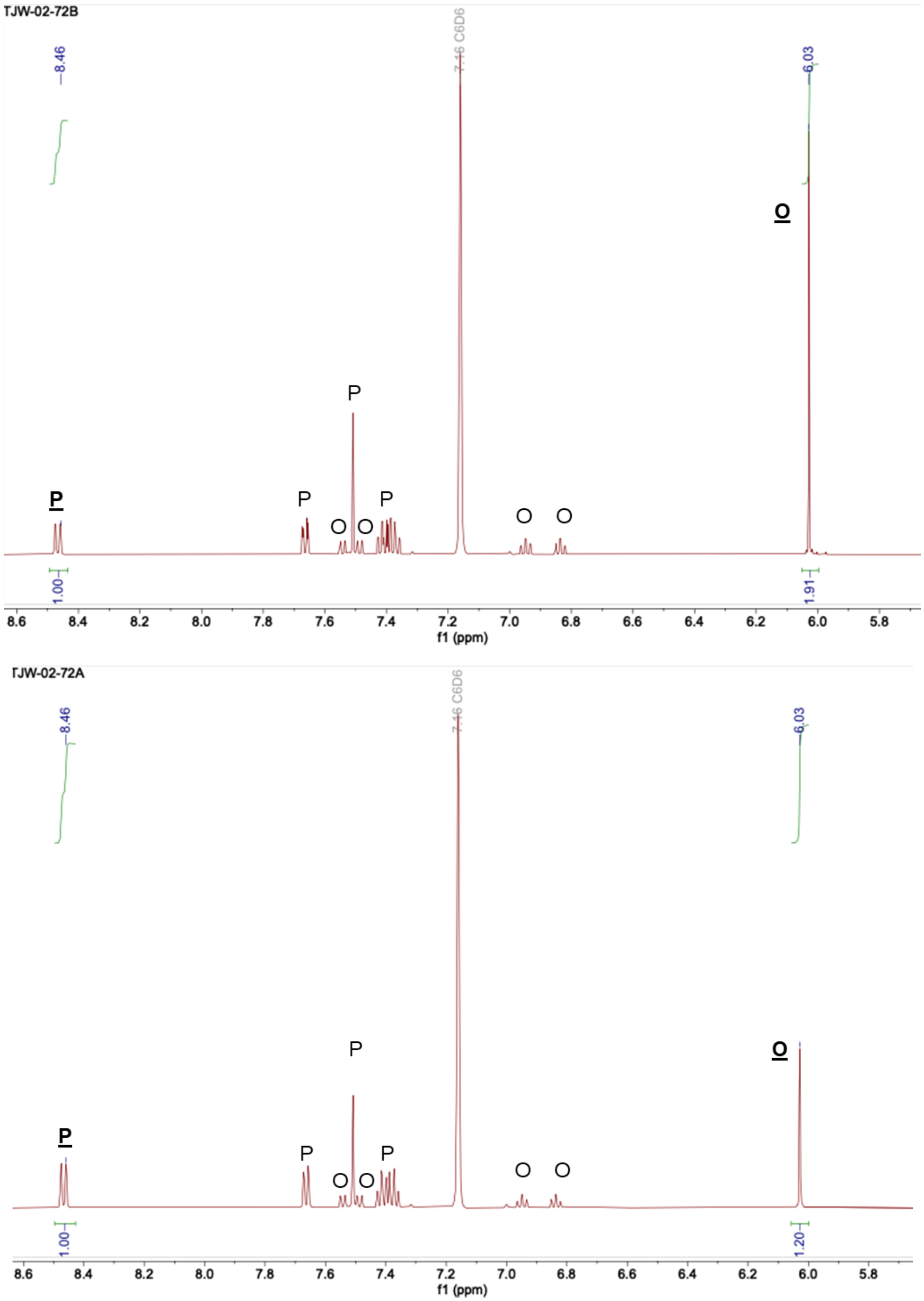

Figure S11: ^{1}H NMR spectra (500 MHz, C_6D_6) of Cp_2Ti(OBET) doped with phenanthrene before (top) and after (bottom) 100 seconds of photolysis under 4 RPR 4190 bulbs. Peaks marked with "P" and "O" correspond to protons on phenanthrene and Cp_2Ti(OBET), respectively. The integrated peaks with a bolded and underlined label were used for all mathematical calculations associated with the Φ_{decomp} experiments.

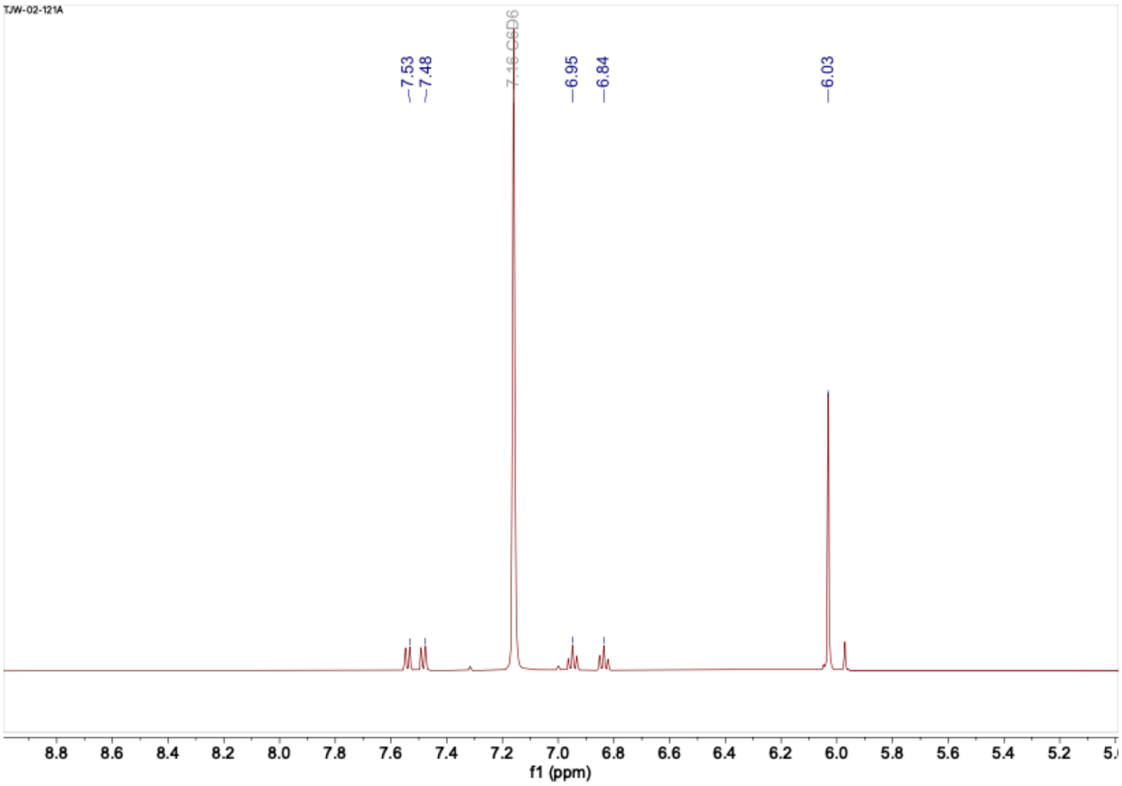

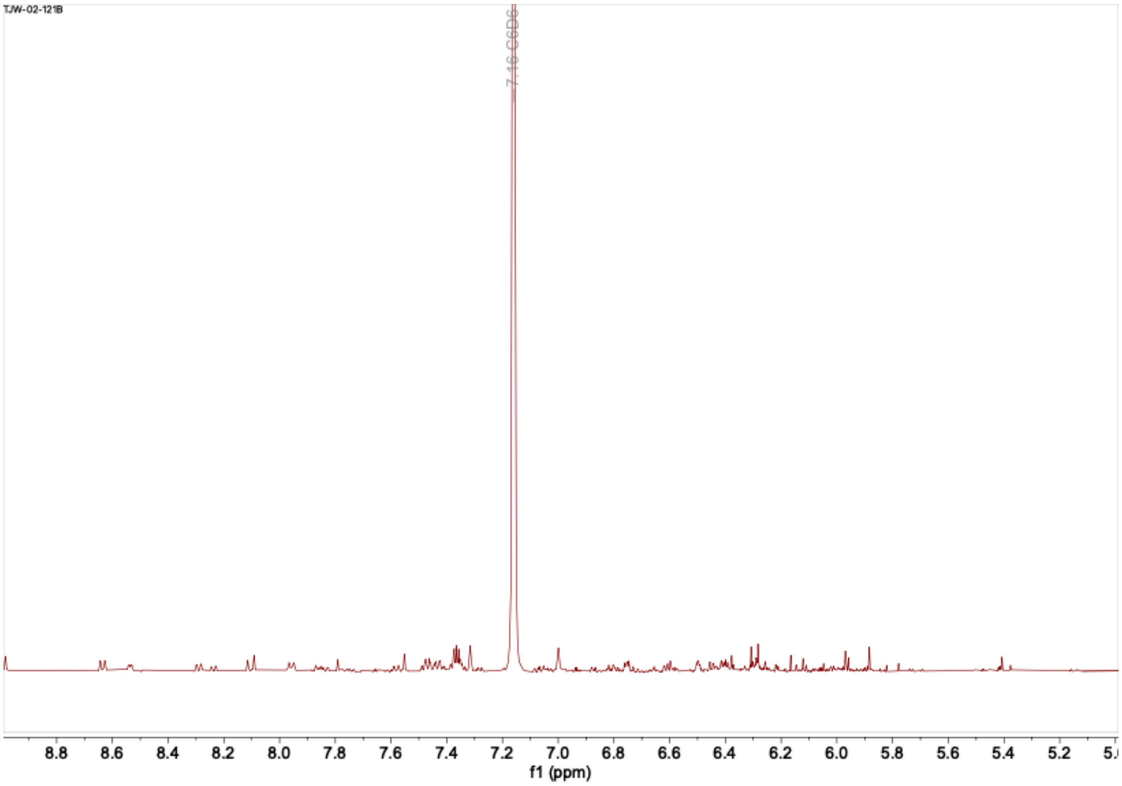

Figure S12: ^{1}H NMR spectra (500 MHz, C_6D_6) of $Cp_2Ti(OBET)$ before (top) and after (bottom) 15 minutes of photolysis under 4 RPR 4190 bulbs.

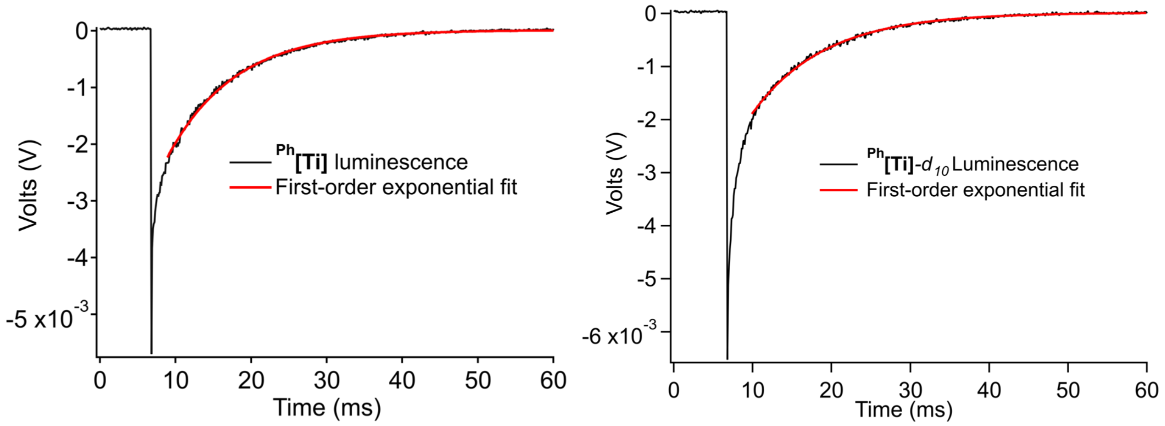

Figure S13: Luminescence decay curves for $Cp_2Ti(C_2Ph)_2$ (left) and $Cp_2Ti(C_2Ph)_2$-d_{10} (right) collected in a 77 K matrix of 2-methyltetrahydrofuran overlaid with first-order exponential fits. λ_{ex} = 367 nm, λ_{em} = 575 nm.

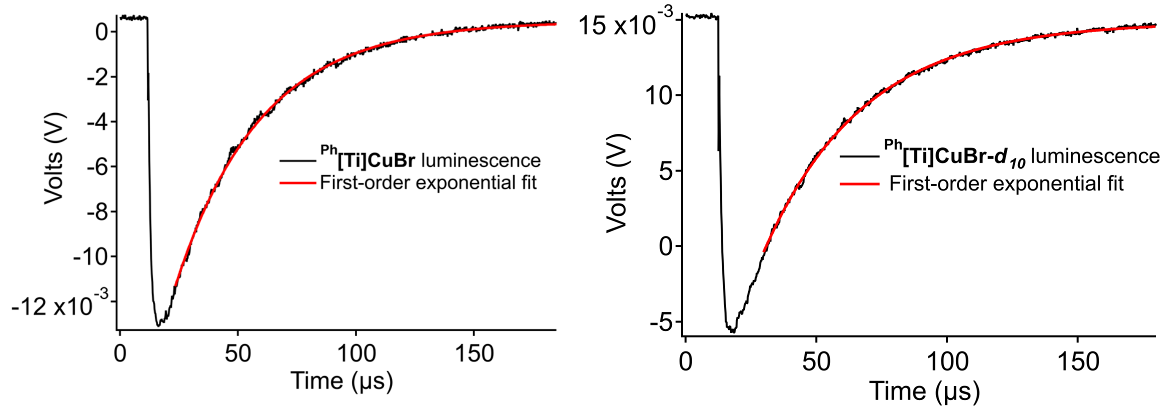

Figure S14: Luminescence decay curves for $Cp_2Ti(C_2Ph)_2CuBr$ (left) and $Cp_2Ti(C_2Ph)_2$-d_{10}CuBr (right) collected in a 77 K matrix of 2-methyltetrahydrofuran overlaid with first-order exponential fits. λ_{ex} = 367 nm, λ_{em} = 715 nm. The lifetime for $Cp_2Ti(C_2Ph)_2CuBr$ was incorrectly reported as 19.4 µs in reference 66 and is corrected herein to be 35.8 µs.

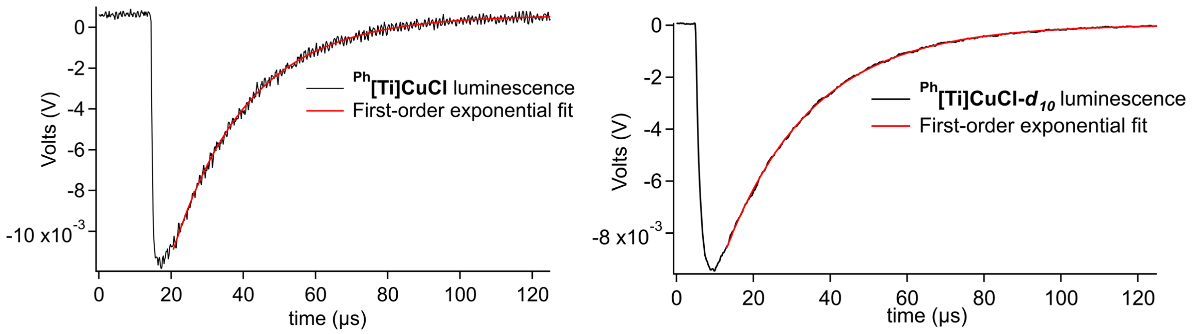

Figure S15: Luminescence decay curves for $Cp_2Ti(C_2Ph)_2CuCl$ (left) and $Cp_2Ti(C_2Ph)_2$-d_{10}CuCl (right) collected in a 77 K matrix of 2-methyltetrahydrofuran overlaid with first-order exponential fits. λ_{ex} = 367 nm, λ_{em} = 706 nm.

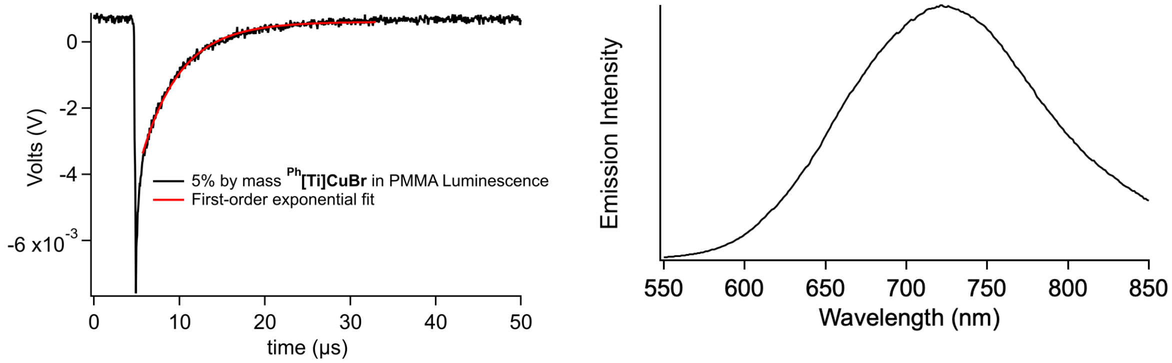

Figure S16: Luminescence decay curve for a 5 wt % film of $Cp_2Ti(C_2Ph)_2CuBr$ (left, λ_{ex}=367 nm, λ_{em}=740 nm) and the plotted emission intensity of a 15 wt % film of $Cp_2Ti(C_2Ph)_2CuBr$ (right, λ_{ex}=460). PMMA films were prepared by the doctor blade method using 150 mg total mass (PMMA + $Cp_2Ti(C_2Ph)_2CuBr$) in 1 mL of CH_2Cl_2.

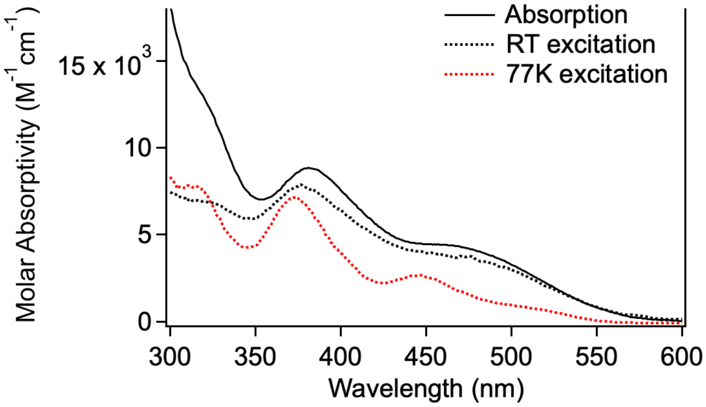

Figure S17: Excitation spectra of $Cp^*{}_2Ti(C_2Ph)_2CuBr$ collected in a room-temperature fluid solution of THF (λ_{em}= 693 nm) and 77 K matrix of 2-methyltetrahydrofuran (λ_{em}= 622 nm) overlaid with the UV-Vis absorption spectrum in RT THF solution.

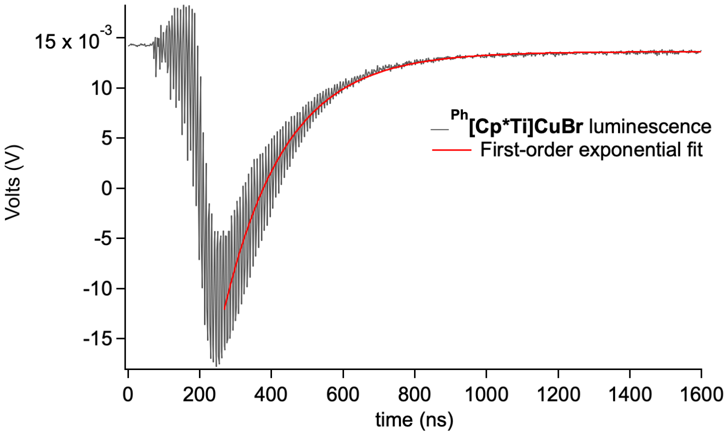

Figure S18. Luminescence decay curve and fit for $Cp^*{}_2Ti(C_2Ph)_2CuBr$ collected in a room-temperature THF solution (λ_{ex} = 367 nm).

Chart S3: Orbital contributions for lowest-energy triplet and singlets of $Cp^{*}_2Ti(C_2Ph)_2CuBr$ using MN15/LANL2DZ//B3LYP/6-311+G(d).

Excited State 1: Triplet-A 1.9304 eV 642.28 nm f=0.0000 <S**2>=2.000
143 ->148 0.13797
146 ->148 0.59609
146 ->153 0.10818
147 ->148 0.30148

Excited State 4: Singlet-A 2.2098 eV 561.07 nm f=0.0036 <S**2>=0.000
143 ->148 0.12766
146 ->148 0.60046
147 ->148 0.31287

Excited State 6: Singlet-A 2.4284 eV 510.57 nm f=0.0107 <S**2>=0.000
141 ->148 -0.10583
142 ->148 -0.10439
144 ->148 0.55490
145 ->148 0.25222
146 ->148 0.11665
147 ->148 -0.22629

Excited State 7: Singlet-A 2.4381 eV 508.54 nm f=0.0203 <S**2>=0.000
142 ->148 -0.14769
144 ->148 -0.38512
145 ->148 0.39835
146 ->148 0.19229
147 ->148 -0.30926

Excited State 9: Singlet-A 2.7440 eV 451.84 nm f=0.0011 <S**2>=0.000
141 ->148 0.62012
141 ->153 0.11782
142 ->148 -0.28698

Excited State 12: Singlet-A 2.8788 eV 430.68 nm f=0.1682 <S**2>=0.000
133 ->148 0.14236
142 ->148 -0.12084
145 ->148 0.39944
146 ->148 -0.22818
147 ->148 0.47961

Excited State 18: Singlet-A 3.3421 eV 370.97 nm f=0.0002 <S**2>=0.000
136 ->149 -0.11254

139 ->149 -0.10169
143 ->150 -0.10739
144 ->149 0.46974
146 ->150 -0.40129
147 ->150 -0.18452

Excited State 19: Singlet-A 3.3813 eV 366.68 nm f=0.0086 <S**2>=0.000
135 ->148 0.22075
143 ->148 0.62811
146 ->148 -0.12595

Excited State 21: Singlet-A 3.4504 eV 359.34 nm f=0.0106 <S**2>=0.000
136 ->148 -0.18961
139 ->148 -0.12320
142 ->149 -0.16195
144 ->152 -0.11751
145 ->149 0.40498
146 ->149 0.22769
147 ->149 -0.34298

Excited State 25: Singlet-A 3.5156 eV 352.67 nm f=0.2075 <S**2>=0.000
136 ->148 0.28599
139 ->148 0.27073
140 ->148 0.48943
145 ->149 0.14810
147 ->149 -0.13827

Excited State 27: Singlet-A 3.5674 eV 347.55 nm f=0.1041 <S**2>=0.000
136 ->148 0.41567
139 ->148 0.15439
140 ->148 -0.40023
141 ->148 0.11163
142 ->148 0.27124
145 ->148 0.11508

Excited State 28: Singlet-A 3.6208 eV 342.42 nm f=0.1162 <S**2>=0.000
136 ->148 -0.23913
139 ->148 -0.11210
140 ->148 0.16877
141 ->148 0.24037
142 ->148 0.51263
145 ->148 0.24046

```
Excited State  30:      Singlet-A      3.6992 eV  335.16 nm  f=0.0005  <S**2>=0.000
    141 ->149         0.43338
    142 ->149        -0.19887
    143 ->152        -0.11102
    146 ->152        -0.40468
    146 ->153        -0.14171
    147 ->152        -0.20357

Excited State  31:      Singlet-A      3.7431 eV  331.24 nm  f=0.0299  <S**2>=0.000
    143 ->149         0.11758
    144 ->150        -0.28542
    146 ->149         0.52872
    147 ->149         0.28312

Excited State  33:      Singlet-A      3.8707 eV  320.32 nm  f=0.0296  <S**2>=0.000
    141 ->149        -0.12590
    143 ->150         0.11926
    144 ->149         0.42564
    146 ->150         0.37669
    147 ->150         0.30083

Excited State  34:      Singlet-A      3.8910 eV  318.64 nm  f=0.0022  <S**2>=0.000
    141 ->149         0.23344
    142 ->149        -0.12015
    142 ->150        -0.11944
    144 ->149         0.17447
    145 ->150         0.35737
    146 ->150         0.27866
    146 ->152         0.24364
    147 ->150        -0.25485

Excited State  35:      Singlet-A      3.9007 eV  317.85 nm  f=0.0011  <S**2>=0.000
    141 ->150         0.43788
    142 ->150        -0.21932
    144 ->152        -0.37688
    144 ->153        -0.13656
    145 ->149        -0.12149
    147 ->149         0.14214

Excited State  41:      Singlet-A      4.0934 eV  302.89 nm  f=0.0032  <S**2>=0.000
    142 ->152        -0.17595
    145 ->152         0.42025
    145 ->153         0.14935
```

146 ->152 0.15399
147 ->152 -0.38142
147 ->153 -0.11978

Excited State 42: Singlet-A 4.1050 eV 302.03 nm f=0.0384 <S**2>=0.000
132 ->148 0.13449
134 ->148 0.21155
135 ->148 0.16355
136 ->148 -0.28960
139 ->148 0.48009
140 ->148 -0.13744
146 ->151 -0.13010

Excited State 43: Singlet-A 4.1141 eV 301.36 nm f=0.0067 <S**2>=0.000
136 ->148 -0.10909
139 ->148 0.12114
141 ->150 -0.13186
144 ->152 -0.31313
144 ->153 -0.10548
146 ->151 0.36626
146 ->154 0.29011
147 ->149 -0.12278
147 ->151 0.17018
147 ->154 0.12990

Excited State 46: Singlet-A 4.1766 eV 296.85 nm f=0.0085 <S**2>=0.000
135 ->148 0.55862
139 ->148 -0.15999
143 ->148 -0.20291
144 ->151 -0.16015

Excited State 51: Singlet-A 4.3088 eV 287.75 nm f=0.1010 <S**2>=0.000
135 ->148 0.17018
141 ->149 0.24823
142 ->149 -0.10812
144 ->151 0.33350
144 ->154 0.26776
145 ->150 -0.19306
146 ->152 0.26580
146 ->153 0.10974
147 ->152 0.10228

```
Excited State  53:      Singlet-A      4.3528 eV  284.84 nm  f=0.0056  <S**2>=0.000
     133 ->149         0.11074
     141 ->150        -0.22625
     142 ->149        -0.16947
     144 ->152        -0.21457
     145 ->149         0.29674
     146 ->149        -0.18165
     147 ->149         0.42789

Excited State  54:      Singlet-A      4.3962 eV  282.03 nm  f=0.0540  <S**2>=0.000
     141 ->152        -0.40466
     141 ->153        -0.14387
     142 ->152         0.17873
     144 ->150         0.45869
     146 ->149         0.15060

Excited State  55:      Singlet-A      4.4447 eV  278.95 nm  f=0.0009  <S**2>=0.000
     141 ->150         0.32757
     142 ->149        -0.12524
     142 ->150        -0.15208
     144 ->152         0.29572
     145 ->149         0.24909
     146 ->151         0.24028
     146 ->154         0.15741
     147 ->149         0.16532
     147 ->151         0.14529
     147 ->154         0.10156

Excited State  59:      Singlet-A      4.5900 eV  270.12 nm  f=0.0011  <S**2>=0.000
     141 ->152         0.23509
     142 ->152        -0.10917
     144 ->150         0.16183
     145 ->151        -0.27661
     145 ->154        -0.21030
     146 ->151        -0.19653
     146 ->154        -0.12233
     147 ->151         0.35298
     147 ->154         0.21598

Excited State  60:      Singlet-A      4.6022 eV  269.40 nm  f=0.0013  <S**2>=0.000
     137 ->148         0.18354
     137 ->151         0.10322
     138 ->148         0.63032
```

Excited State 61: Singlet-A 4.6026 eV 269.38 nm f=0.0263 <S**2>=0.000
137 ->148 0.50943
138 ->148 -0.18973
140 ->149 -0.31856

Excited State 63: Singlet-A 4.6225 eV 268.22 nm f=0.0042 <S**2>=0.000
134 ->149 0.12641
135 ->148 0.12494
137 ->148 0.34349
139 ->149 0.12866
140 ->149 0.50275

Excited State 65: Singlet-A 4.7108 eV 263.19 nm f=0.0933 <S**2>=0.000
122 ->148 -0.13717
128 ->148 0.32285
133 ->148 0.39805
137 ->148 0.11123
141 ->151 0.20780
141 ->154 0.16660
145 ->148 -0.11340
147 ->150 0.10861
147 ->152 -0.12665

Excited State 67: Singlet-A 4.7630 eV 260.31 nm f=0.0492 <S**2>=0.000
132 ->148 0.26754
134 ->148 0.50219
136 ->148 0.10055
139 ->148 -0.22898
145 ->150 -0.11520
147 ->150 -0.18764

Excited State 68: Singlet-A 4.7664 eV 260.12 nm f=0.0271 <S**2>=0.000
132 ->148 0.10757
134 ->148 0.21068
140 ->149 -0.15651
141 ->151 -0.15388
141 ->154 -0.13087
142 ->150 -0.14154
145 ->150 0.23933
146 ->150 -0.20446

147 ->150 0.41312
147 ->152 0.11531

Excited State 70: Singlet-A 4.7927 eV 258.70 nm f=0.0012 <S**2>=0.000
128 ->148 -0.20695
133 ->148 -0.26932
141 ->151 0.34803
141 ->154 0.26082
142 ->151 -0.15577
142 ->154 -0.12459
145 ->150 0.12103
147 ->150 0.17944
147 ->152 -0.17923

Excited State 73: Singlet-A 4.9352 eV 251.22 nm f=0.4513 <S**2>=0.000
131 ->148 0.21165
135 ->148 0.14946
141 ->149 -0.19447
141 ->151 -0.12263
144 ->151 0.35840
144 ->154 0.13005
145 ->150 0.23303
146 ->153 -0.23590
147 ->153 -0.11622

Excited State 74: Singlet-A 4.9403 eV 250.96 nm f=0.0505 <S**2>=0.000
133 ->152 0.11572
141 ->151 0.14707
141 ->154 0.10769
142 ->152 -0.15976
145 ->152 0.37138
146 ->152 -0.18278
147 ->152 0.42297
147 ->153 -0.10487

Excited State 75: Singlet-A 4.9789 eV 249.02 nm f=0.0030 <S**2>=0.000
130 ->148 -0.10644
132 ->150 -0.10446
134 ->150 0.14839
139 ->150 0.16145
140 ->150 0.60785

Excited State 76: Singlet-A 5.0008 eV 247.93 nm f=0.1884 <S**2>=0.000

```
   130 ->148        -0.14830
   132 ->148        -0.15018
   134 ->148         0.11447
   141 ->152        -0.11993
   143 ->149         0.18590
   143 ->151        -0.11748
   144 ->150        -0.11788
   144 ->153        -0.15576
   145 ->151         0.21720
   146 ->149        -0.10871
   146 ->154        -0.13748
   147 ->151         0.40149
   147 ->154        -0.10037

Excited State  77:      Singlet-A      5.0153 eV  247.21 nm  f=0.0687  <S**2>=0.000
   132 ->148        -0.27072
   134 ->148         0.12389
   135 ->149         0.12761
   140 ->152        -0.16658
   142 ->151         0.10827
   143 ->149         0.34301
   145 ->149         0.10059
   145 ->151        -0.24636
   146 ->151         0.14695
   147 ->151        -0.23882

Excited State  78:      Singlet-A      5.0351 eV  246.24 nm  f=0.0060  <S**2>=0.000
   130 ->148        -0.19990
   132 ->148         0.40717
   134 ->148        -0.18505
   140 ->152         0.18361
   143 ->149         0.22503
   144 ->153        -0.12486
   145 ->151        -0.13020
   146 ->151         0.12355
   146 ->154        -0.16235

Excited State  79:      Singlet-A      5.0702 eV  244.53 nm  f=0.2410  <S**2>=0.000
   131 ->148         0.31563
   136 ->151         0.11333
   140 ->149        -0.11223
   141 ->149         0.10862
   144 ->154        -0.29079
```

145 ->150 -0.15675
146 ->152 0.19238
146 ->153 -0.21300
147 ->153 -0.22923

Excited State 80: Singlet-A 5.0919 eV 243.49 nm f=0.0268 <S**2>=0.000
130 ->148 0.27391
135 ->151 0.10460
140 ->150 0.13707
143 ->149 0.39709
144 ->153 0.16800
145 ->149 0.13377
146 ->149 -0.10408
146 ->151 -0.18065
146 ->154 0.13562
147 ->153 -0.11480
147 ->154 0.13907

Chart S4. Frontier orbitals corresponding to the lowest-energy triplet and singlets for $Cp^{*}{}_2Ti(C_2Ph)_2CuBr$ using B3LYP/6-311+G(d).[a]

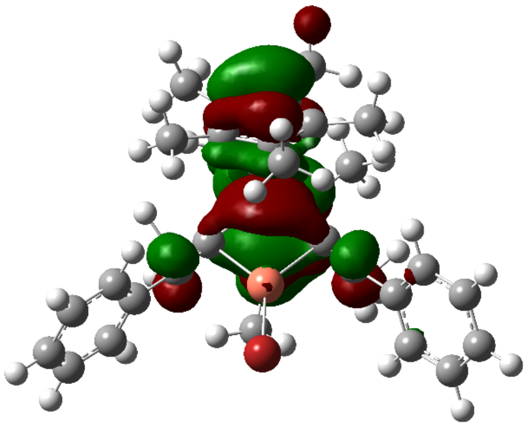	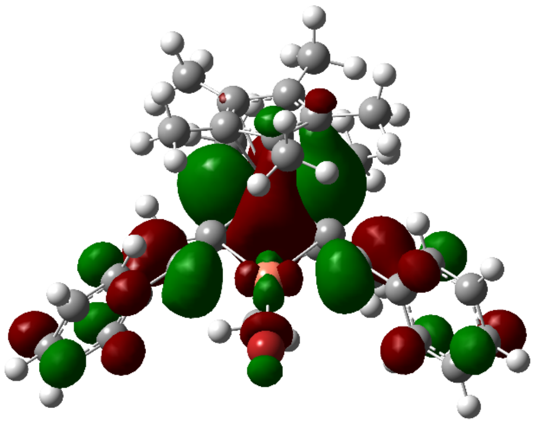
LUMO+1 (MO=149, Energy=-0.03082)	LUMO (MO=148, Energy=-0.08148)

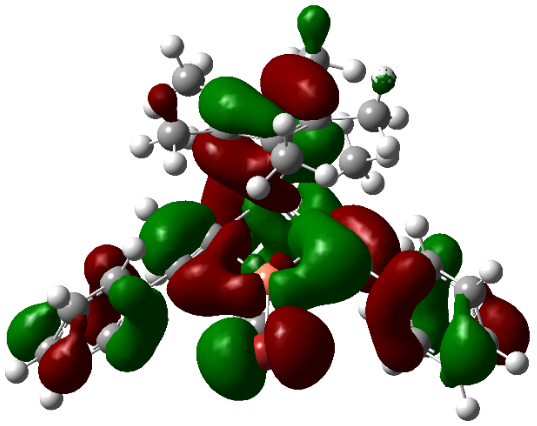	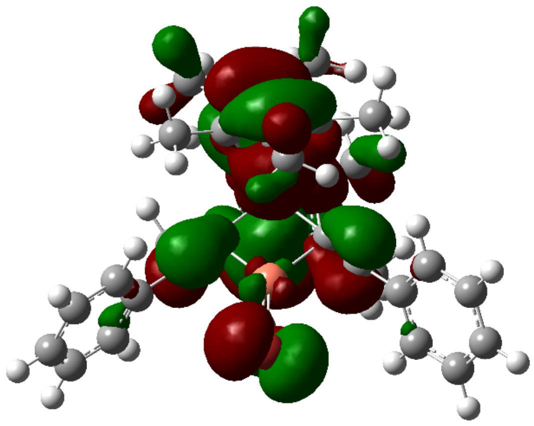
HOMO (MO=147, Energy=-0.24708)	HOMO-1 (MO=146, Energy=-0.25089)
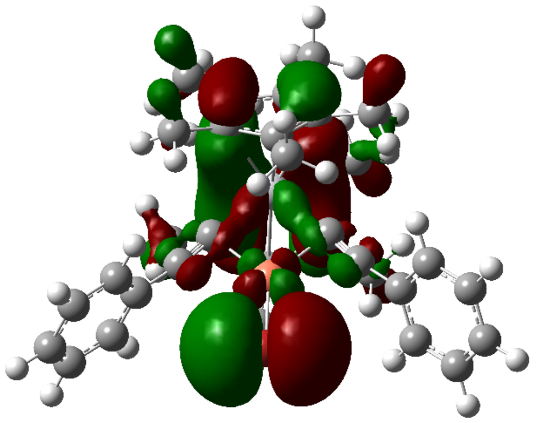	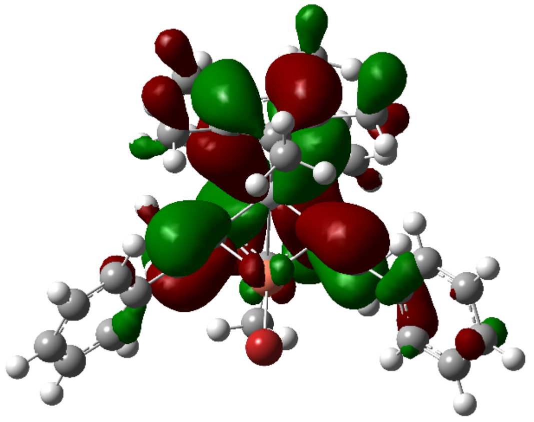
HOMO-2 (MO=145, Energy=-0.25817)	HOMO-3 (MO=144, Energy=-0.26031)
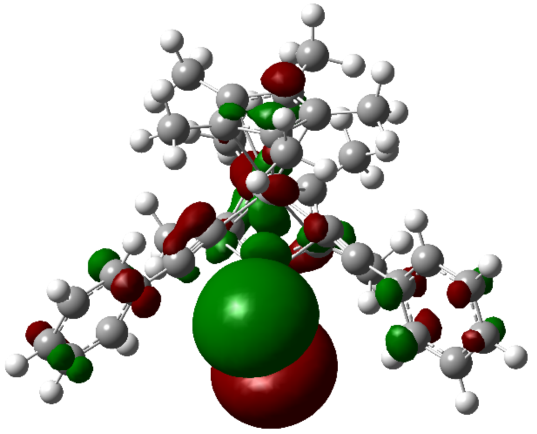	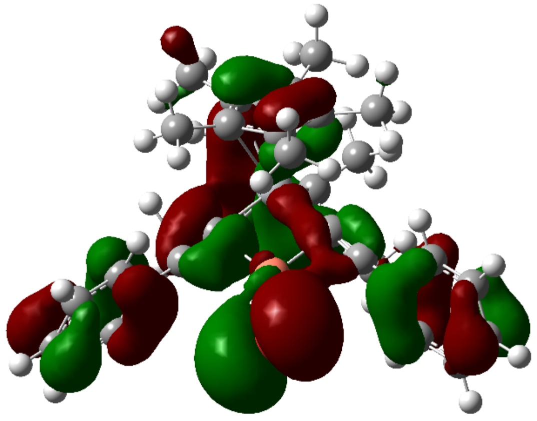
HOMO-4 (MO=143, Energy=-0.26582)	HOMO-5 (MO=142, Energy=-0.26837)

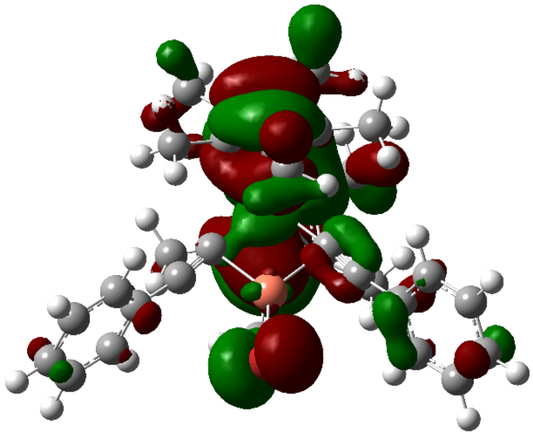	
HOMO-6 (MO=141, Energy=-0.26883)	

[a]A default isovalue of 0.02 is used for all surfaces.

Table S2: Mulliken population analysis of lowest energy singlet and triplets of $Cp*_2Ti(C_2Ph)_2CuBr$ using MN15/LANL2DZ//B3LYP/6-311+G(d).

ES[a]	Osc.	λ (nm)	Ti	Cp*	C2	Ph	Cu	Br
1	-	642	12-->57	54-->7	14-->16	6-->14	3-->5	11-->1
(T)			(45)	(-47)	(2)	(8)	(2)	(-10)
					H-1->LUMO (71%), HOMO->LUMO (18%)			
4	0.0036	561	12-->58	53-->7	14-->16	6-->12	4-->5	11-->1
(S)			(46)	(-46)	(2)	(6)	(1)	(-10)
					H-1->LUMO (72%), HOMO->LUMO (20%)			
6	0.0107	511	14-->58	51-->7	16-->16	6-->12	4-->5	8-->1
(S)			(44)	(-44)	(0)	(6)	(1)	(-7)
					H-3->LUMO (62%), H-2->LUMO (13%), HOMO->LUMO (10%)			
7	0.0203	509	11-->58	45-->7	13-->16	7-->12	6-->5	18-->1
(S)			(47)	(-38)	(3)	(5)	(-1)	(-17)
					H-3->LUMO (30%), H-2->LUMO (32%), HOMO->LUMO (19%)			
9	0.0011	452	15-->57	59-->7	3-->16	9-->14	2-->5	11-->1
(S)			(42)	(-52)	(13)	(5)	(3)	(-10)
					H-6->LUMO (77%), H-5->LUMO (16%)			
12	0.1682	431	6-->58	38-->7	13-->16	13-->12	9-->5	20-->1
(S)			(52)	(-31)	(3)	(-1)	(-4)	(-19)
					H-2->LUMO (32%), H-1->LUMO (10%), HOMO->LUMO (46%)			

[a] Triplet transitions are denoted with a “T” and singlet transitions are marked with an “S”.
